# A randomized controlled trial protocol comparing low-calorie Mediterranean and low-carbohydrate diets for diabetes remission in individuals with type 2 diabetes in northern Lebanon: an intervention mapping–based approach

**DOI:** 10.3389/fpubh.2026.1787980

**Published:** 2026-04-08

**Authors:** Janot J. Ayoub, Suzan A. Haidar, Ellen E. Blaak, Dennis De Ruijter

**Affiliations:** 1 Department of Health Promotion, NUTRIM, Institute of Nutrition and Translational Research in Metabolism, Maastricht University Medical Centre, Maastricht, Netherlands; 2 Department of Nutrition, Faculty of Nutrition and Health Sciences, Lebanese International University (LIU), Beirut, Lebanon; 3 Lebanese International University (LIU), Beirut, Lebanon; 4 Department of Human Biology, NUTRIM, Institute of Nutrition and Translational Research in Metabolism, Maastricht University, Maastricht, Netherlands; 5 Department of Health Promotion, NUTRIM, Institute of Nutrition and Translational Research in Metabolism, Maastricht University, Maastricht, Netherlands

**Keywords:** behavior change, diabetes remission, intervention mapping protocol, lifestyle modification, Mediterranean diet, mobile health application, type 2 diabetes mellitus, weight loss

## Abstract

**Background:**

Type 2 diabetes mellitus (T2DM) is increasing worldwide, largely due to obesity and unhealthy lifestyle behaviors. Weight-loss–induced diabetes remission offers a promising approach to reducing complications and improving quality of life. Addressing this effectively requires a comprehensive approach that targets behavioral and environmental determinants and is guided by the Intervention Mapping (IM) protocol.

**Objective:**

This paper describes the protocol for a randomized controlled trial comparing two low-calorie dietary approaches for achieving diabetes remission in adults with T2DM, using an IM-based intervention framework.

**Methods:**

This is a two-phase, multi-center, parallel-group randomized controlled trial conducted in primary medical centers in Northern Lebanon. A total of 50 adults aged 18–65 years, with a maximum diabetes duration of 6 years and treated with oral antidiabetic medications, are enrolled. Participants are randomly assigned to follow either a low-calorie Mediterranean-style diet or a low-calorie, low-carbohydrate diet. The study consists of a 6-month weight loss phase developed using the six steps of the (IM) protocol, followed by a 6-month maintenance phase supported by a mobile health application, resulting in a total follow-up duration of 12 months. The primary outcome is diabetes remission at 6 months (HbA_1c_ < 6.5% with or without discontinuation of medication). Secondary outcomes include sustained remission at 12 months, changes in weight and body composition, glycemic control, lipid profile, sleep quality, physical activity, psychological stress, and inflammatory markers. Secondary outcomes will be assessed at baseline and every 3 months and analyzed exploratorily.

**Discussion:**

Sustained weight loss is essential for achieving diabetes remission and reducing metabolic complications; however, long-term success depends on realistic and maintainable behavioral changes. This protocol applies the IM framework to systematically develop a culturally relevant, theory-based intervention tailored to individuals with T2DM in Lebanon. By combining dietary strategies with behavioral support and mobile health technology, the study addresses barriers to long-term adherence. The findings are expected to inform the design of scalable, context-specific interventions for diabetes management in similar low- and middle-income settings.

**Clinical trial registration:**

https://www.isrctn.com/ISRCTN99204002, Identifier ISRCTN99204002.

## Introduction

Diabetes represents one of the most rapidly escalating global health challenges, significantly impacting individuals, families, and communities. As a serious, long-term condition, it is among the ten leading causes of adult mortality, claiming approximately 6.7 million deaths worldwide in 2021 (1) and affecting 9.3% of the global population in 2019 (2) and 10.5% in 2022 (3), a figure expected to rise to 10.9% by 2045 (2). The Middle East and North Africa regions have the highest prevalence, with rates projected to reach 13.9% by 2045 (2, 4). In Lebanon, 7.8% of adults aged 20–79 live with Type 2 Diabetes Mellitus (T2DM) (5) which accounts for approximately 90 to 95% of all diabetes cases (6). T2DM contributes to premature mortality, increased healthcare costs, and reduced quality of life (2, 7, 8). In addition to its clinical burden, T2DM imposes substantial financial costs estimated at 760 billion dollars globally in 2019 (2) and affects productivity through increased absenteeism (8, 9). According to the American Diabetes Association (ADA), healthcare spending per person with diabetes is 2.3 times higher than for those without the disease, with a substantial portion of costs linked to diabetes-related comorbidities (10). In the Middle East, the mean hospitalization cost for individuals with diabetes reached approximately $1,436, surpassing the International Diabetes Federation’s global per-person cost estimate (11).

Obesity is the leading cause of T2DM and a key contributor to its growing prevalence (12). This condition has become increasingly prevalent across the globe, raising significant concerns that the World Health Organization has termed this issue an “escalating global epidemic” (3). It is one of the most visible health problems impacting people of all ages and socioeconomic backgrounds worldwide (13). In 2022, approximately 2.5 billion adults were classified as overweight, with 890 million living with obesity. Projections suggest that by 2050, this number could rise to 3.8 billion adults experiencing overweight and obesity (13). The growing burden of these conditions is closely linked to a higher incidence of T2DM and various cardiometabolic complications. This is primarily due to abdominal fat, which can lead to insulin resistance, pancreatic dysfunction, and increased inflammation, ultimately contributing to greater morbidity and mortality rates (14, 15).

For years, medical treatments and lifestyle modifications have been the cornerstone of diabetes control, promoting weight loss, physical activity (PA), and a healthy diet (16–18).

Since obesity is a significant risk factor for T2DM and its associated complications, weight loss is strongly recommended to enhance insulin sensitivity and delay the need for exogenous insulin 19. Evidence shows that 10–15% weight loss, achieved through bariatric surgery or intensive dietary interventions, can induce diabetes remission (18–22).

Dietary modification, therefore, represents a practical and sustainable approach to achieve such weight reduction and metabolic improvement. Among nutritional approaches, the Low Carbohydrate (LC) diet and the Mediterranean Diet (MD) have shown promising improvements in metabolic profiles, often linked to weight loss (23–26), and have been investigated for T2DM management and remission (27, 28). LC diets have a carbohydrate content of less than 45% and are further categorized into three groups: very LC or ketogenic LC, which contains less than 10% of carbohydrates, LC diet, which has a range of 10 to 26% of carbs or less than 130 g of carbohydrates per day, and moderately LC, which includes 26 to 45% of carbohydrates (27, 29, 30). A systematic review examining the dose-effect of carbohydrate restriction found that moderate carbohydrate restriction (26–45%) resulted in reductions in blood glucose levels, HbA1c, and blood lipids over six months when compared to high-carbohydrate diets (31). However, these effects became insignificant after six months, particularly with very low-carbohydrate diets, likely due to low adherence (31). On the other hand, research indicates that a range of dietary patterns, including traditional MD, appears suitable for translating nutritional recommendations into practical advice for individuals with diabetes (32, 33), a finding previously validated in a systematic review comparing several dietary approaches for the management of T2DM (34). In a 16-week weight-loss study, patients with T2DM who followed an LC or an MD diet experienced significant reductions in blood sugar, fat levels, and glycated hemoglobin (HbA1c), with the LC diet showing slightly better results because it led to greater weight loss than the MD diet (35). However, the restrictive nature of the LC diet, which contains less than 130 g of carbs, led to cravings and difficulty maintaining dietary variety, factors that make the MD easier for people to adhere to over time and a better option for managing T2DM in the long run (34, 35).

In 2021, the ADA defined diabetes remission as maintaining an HbA1c < 6.5% for at least three months without glucose-lowering medication (36, 37). However, achieving remission is often hindered by adherence challenges (38) such as poor health literacy, obesogenic environment, poor self-esteem, ambivalence, and socioeconomic factors, particularly in low-resource settings like Lebanon, where socioeconomic status and limited health awareness are common barriers (39).

In this trial, the number of participants achieving remission (HbA1c < 6.5%) without medication will be reported separately from those who achieve HbA1c < 6.5% while continuing pharmacotherapy. This approach was adopted to account for potential reluctance among physicians to discontinue cardiometabolic medications in routine clinical practice. Concerns about rebound hyperglycemia, patient destabilization, and the risk of adverse outcomes may limit medication withdrawal (40). Furthermore, delays in deprescribing may also stem from the absence of well-established guidelines, which can contribute to cautious prescribing behavior, particularly in settings characterized by high levels of daily stress and healthcare system instability (41).

Managing diabetes complications and achieving remission, hence, requires a multidisciplinary team (42). Physicians play a crucial role in communicating the potential for diabetes control, specifically the ability to achieve glycemic stability, delay disease progression, and, in some cases, induce remission through dietary and lifestyle modifications (43). By framing these possibilities in a patient-centered manner and facilitating referrals to dietitians for sustained support, they help initiate and sustain behavior change. Given the personal and environmental barriers to T2DM management (44–46), tailored interventions are essential. The Intervention Mapping (IM) framework provides a flexible and systematic structure for developing such interventions, ensuring that programs are both theory-driven and evidence-based while being adapted to the Lebanese context (47).

The present study aims to systematically document the development process for designing a two-arm dietary intervention using the IM protocol. The trial is designed to compare the effects of a calorie-restricted MD diet and a calorie-restricted LC diet on remission of diabetes. In addition to the remission outcome, the trial examines changes in cardiometabolic risk factors during the 12-month follow-up period.

## Methodology

This manuscript presents the protocol of a randomized clinical trial and adheres to the SPIRIT 2013 statement, which promotes transparency and methodological rigor in the reporting of clinical trial protocols (48). While SPIRIT provides the framework for describing the trial design and procedures, the intervention tested in this study was developed using the IM approach. The key SPIRIT components are outlined below, followed by a description of the intervention development process based on the IM framework.

### Study design

This is a two-phase, multicenter, parallel-group randomized controlled trial conducted in primary medical centers in Northern Lebanon. In the first phase, participants are randomly assigned to a low-calorie MD-style diet or a low-calorie LC diet (<130 g/day) and followed for 6 months, which is the weight-loss phase. Following this phase, a 6-month maintenance phase is supported by a mobile health application. The study protocol was approved by the Lebanese International University Institutional Review Board (LIUIRB-230817-JA-295) and registered in the ISRCTN registry (ISRCTN99204002). It was developed and reported in accordance with the Standard Protocol Items: Recommendations for Interventional Trials (SPIRIT) 2025 checklist, as outlined in Appendix S4.

### Eligibility

Eligible participants are adults aged 18–65 years with a diagnosis of T2DM, a body mass index ≥25 kg/m^2^, treated exclusively with oral antidiabetic medications, and with a disease duration of six years or less.

Participants are excluded if they are treated with insulin therapy, have a history of bariatric surgery, are pregnant or breastfeeding, have chronic liver or kidney disease, have an active malignancy, or have severe psychiatric disorders.

### Sample size calculation

Sample size estimation was based on the primary outcome of diabetes remission at 6 months, defined as HbA1c < 6.5% with or without discontinuation of glucose-lowering medication, a definition used in a large meta-analysis (27). The same meta-analysis reported that LC diets (<130 g/day) were associated with a remission rate of approximately 57% at 6 months.

For the MD group, a remission rate of approximately 15% at 12 months was reported in medication-naïve individuals (49). Given that remission rates are known to be lower among individuals receiving glucose-lowering therapy, as demonstrated in large observational cohorts (50, 51), this supports a conservative estimate for the present study population, which includes participants treated initially with oral antidiabetic medications. Furthermore, as no large randomized trials have reported diabetes remission at 6 months following an MD, and considering evidence from long-term intervention studies showing a decline in remission rates over time, including the DiRECT (52) and Look AHEAD (53) trials, a conservative remission rate of 15% at 6 months was assumed for the MD in the present trial.

It should be acknowledged that the remission estimates used for the sample size calculation were derived from studies conducted in different medication contexts. In both cases, remission was based on the same glycemic threshold (HbA1c < 6.5%). However, the estimate for the MD was derived from a study conducted in medication-naïve individuals, whereas the remission estimate for the LC diet was obtained from studies in which participants were receiving glucose-lowering therapy and remission was defined irrespective of medication use. Therefore, the two estimates are not fully comparable. Nevertheless, in the present trial, remission is defined consistently for both intervention arms as HbA1c < 6.5% regardless of medication use. In the absence of randomized trials reporting remission following an MD in medicated populations, the 15% rate was retained as a conservative estimate for the power calculation.

Using these expected remission rates (57% vs. 15%) and a two-sided *α* level of 0.05 with 80% power, the required sample size was estimated at 38 participants. To account for an anticipated attrition rate of approximately 20%, a total sample size of 50 participants was targeted.

### Randomization

Randomization (by minimization) was performed in the two arms using the computer-generated program “QMinim,” which ensures balance across pre-specified covariates, including age, sex, diabetes duration, and baseline BMI. The allocation sequence was generated by a research assistant who is not involved in enrolling participants or delivering the interventions.

### Intervention

Participants are randomly allocated to one of two dietary intervention arms: a low-calorie MD or a low-calorie LC diet. The intervention is delivered in two sequential phases: an initial weight-loss induction phase followed by a maintenance phase.

#### Phase 1: weight-loss induction (0–6 months)

During Phase 1, participants are randomly divided into two groups. One group follows an LC diet, consuming less than 130 grams of carbohydrates per day, while the other group follows an MD. Both groups receive personalized dietary counseling based on the six steps of the IM framework. The intervention aims to promote weight loss (10–15% of body weight) and enhance diabetes self-management through structured nutrition education, goal setting, behavioral strategies, and regular follow-ups with trained dietitians to achieve remission of diabetes.

Both groups receive comparable frequency and intensity of counseling sessions to ensure standardized behavioral support, differing only in dietary composition. Educational materials and personalized meal plans are provided in accordance with the assigned diet.

#### Phase 2: maintenance phase (6–12 months)

During Phase 2 (6–12 months), a mobile health application will be implemented to support weight maintenance and sustained adherence. The application will enable dietitians to update clinical data and monitor participants’ dietary intake, physical activity, and adherence patterns. Participants will receive tailored meal examples, weekly reminders promoting dietary compliance, physical activity, and stress management, and will log in their 24-h intake and exercise sessions. A secure chat feature will facilitate ongoing communication and individualized feedback, allowing early identification and management of potential adherence barriers.

#### Intervention fidelity

A standardized counseling manual will be used by all dietitians, including structured questionnaires and checklists to ensure consistency in intervention delivery. Dietitians will undergo dedicated training sessions to standardize and harmonize the protocol implementation. Participants will receive individualized counseling sessions and will report dietary adherence using repeated 24-h dietary recalls and food records.

Detailed descriptions of dietary prescriptions, counseling content, and behavioral strategies are provided in the IM Step 4 section.

### Study procedures

Upon enrollment, participants will undergo baseline assessment, including anthropometric measurements, biochemical testing, and completion of standardized questionnaires. Each participant will have an individual study file containing anthropometric, biochemical, and body composition data. Standardized questionnaires will be used to collect demographic and lifestyle information and to assess diabetes-related knowledge, dietary and medication adherence, stress, sleep quality, and PA levels. Participants will attend monthly follow-up visits with dietitians, and laboratory assessments will be performed every three months throughout the trial.

### Outcomes

The primary outcome is diabetes remission at 6 months (HbA_1c_ < 6.5% with or without diabetes medication).

Secondary outcomes include sustained remission at 12 months, changes in body weight and body composition, glycemic control (fasting glucose, HbA1c, fasting insulin), lipid profile, sleep quality, PA, psychological stress, and inflammatory markers. Secondary outcomes will be assessed at baseline and every 3 months and analyzed exploratorily.

### Statistical methods

All analyses will be conducted according to the intention-to-treat principle. Baseline demographic and clinical characteristics will be summarized descriptively by randomized group using means ± standard deviations or medians (interquartile ranges) for continuous variables and frequencies and percentages for categorical variables. In accordance with SPIRIT recommendations, no formal statistical testing will be performed for baseline comparisons.

The primary outcome, diabetes remission—defined as HbA1c < 6.5% irrespective of glucose-lowering medication use—will be analyzed using logistic regression models or generalized mixed-effects logistic models when assessed longitudinally, with intervention group as the primary predictor. Remission prevalence will be reported overall and stratified by medication status (remission achieved without medication and remission achieved while continuing pharmacotherapy). Effect estimates will be presented as odds ratios with 95% confidence intervals.

Secondary outcomes will include changes in metabolic parameters (body weight, BMI, lipid profile, glycemic profile, inflammatory markers, and indices of insulin sensitivity) as well as behavioral and physiological outcomes (PA, sleep quality, and stress). Continuous outcomes will be analyzed using linear mixed-effects models with fixed effects for intervention group, time, and group-by-time interaction, and a random effect for participants to account for repeated measurements. The group-by-time interaction term will be used to assess differential effects between the two dietary interventions.

The effectiveness of the educational component will be evaluated by examining the association between education exposure or knowledge change and the primary and secondary outcomes using multivariable regression models adjusted for relevant baseline covariates.

Missing data will be handled using appropriate statistical methods where applicable. Statistical significance will be set at *p* < 0.05. All analyses will be performed by a blinded statistician using standard statistical software.

## Development of the IM protocol

The intervention was systematically developed using the IM framework, a theory- and evidence-based approach for planning health promotion programs. IM provides a structured process that integrates behavioral theory, empirical evidence, and stakeholder input to design interventions targeting modifiable determinants of health behaviors. The framework comprises six sequential steps that guide the development, implementation, and evaluation of complex interventions (54).

1) Logic model of the program
2) Logic model of change
3) Program design
4) Program production
5) Program implementation
6) Evaluation plan

Each step builds upon the previous one, making the process iterative rather than strictly linear. The IM framework involves a series of specific tasks within each step, which are described in detail in the following sections.

### Step 1: logic model of the problem

This step aims to assess and describe the health problem, its related behaviors, and environmental conditions, as well as their associated determinants, for the at-risk population. The following tasks were required to complete step 1: establish a planning group, conduct a needs assessment, and create a logic model.

To systematically address these tasks, the following procedures were undertaken during step 1. First, we designed a planning group comprising six dietitians with expertise in therapeutic nutrition. The planning group was responsible for implementing the study protocol from recruiting patients with T2DM to education, follow-up, and weight management. Then, for the needs assessment, a systematic review was conducted to identify determinants of adherence to the MD in Mediterranean countries 56. This review provided evidence-based insights into the cultural, behavioral, and environmental factors that influence dietary adherence. In parallel, additional studies were consulted to examine the knowledge and practices of patients with T2DM 57,58, the effect of implementing nutrition guidelines on diabetes management 59, and to evaluate the stress levels related to diabetes management 60. The combined findings from these sources informed the development of a discussion guide for focus groups, which are described in detail below, ensuring that the discussion topics reflected evidence from the international literature while remaining tailored to the Lebanese context.

Following this, and because qualitative methods such as focus groups are widely recommended in IM for capturing stakeholder perspectives and contextualizing behavioral determinants (54) we conducted three stakeholder focus groups to complement the literature review. These groups were selected to reflect the multidisciplinary and patient-centered approach required in T2DM management, in which physicians, dietitians, and patients work collaboratively to optimize care, prevent complications, and improve quality of life (55). The focus group interviews were conducted by a trained dietitian from the research team using a semi-structured guide with predefined questions. The first included 10 endocrinologists and explored barriers and facilitators to integrating nutrition therapy into standard diabetes management. The second group consisted of 10 patients with T2DM, who were brought together to investigate their perceptions of the disease, self-management strategies, adherence to treatment, expectations for family support, and their nutritional and medical care needs. The third comprised 10 dietitians and examined the challenges they encounter in providing diabetes nutrition counseling, the dietary regimens they prescribe, patient engagement levels, and the frequency of physician referrals for dietetic services.

The next task was to create a logic model that illustrates the problem, to guide the planning team in addressing the key health challenges (54).

#### Findings from literature review

In Lebanon, patients with T2DM often demonstrate inadequate self-management of the disease as they lack knowledge, confidence, and self-efficacy in controlling their condition and preventing the associated complications (56, 57). Literature indicates multiple barriers to effective management, including:

- Dietary-related barriers: unhealthy dietary habits and insufficient knowledge about diet therapy, leading to a failure in achieving weight loss (56).
- Treatment adherence issues: lack of medication adherence (58, 59), and low referral rates to dietitians, often due to budgetary constraints and limited availability of dietetic services (57, 60).
- Lifestyle factors: inadequate sleep (57), physical inactivity, and smoking (58, 61).
- Psychosocial and economic challenges: high stress levels, often linked to reduced self-efficacy and increased risk of depression (62), as well as poor economic status, limiting access to medical care (57).

Non-adherence to medical and dietary recommendations was primarily seen in patients with high body mass index (BMI), those with lower levels of education, smokers, and those who are physically inactive (58, 61). Notably, referrals to dietetic services were more common when dietetic clinics were located within medical centers, possibly reflecting the influence of institutional policies promoting multidisciplinary care (63). Moreover, dietary interventions have been shown to improve clinical parameters of T2DM and alleviate its complications after a 12-month follow-up period (64).

#### Findings from focus groups

The interview with endocrinologists (*n* = 10) revealed that T2DM is a common condition in their practice. While medications remain central to management, they highlighted the crucial role of diet and weight loss in improving outcomes. Reported barriers to adherence included financial constraints, low education, and unhealthy lifestyles. Although six out of 10 endocrinologists routinely refer patients to dietitians, others do so only when glycemic control is poor. Most endorsed LC diets, regular self-monitoring, and agreed that T2DM remission is achievable with full adherence and weight loss.

On the other hand, patients with T2DM (*n* = 10) described their condition as moderately to highly severe and reported relying primarily on medication. While they recognized the benefits of diet and weight loss, they lacked adequate dietary guidance. Although seven out of 10 patients attempted to lose weight on their own since their diagnosis with diabetes, stress and financial constraints hindered their adherence to a self-designed dietary program. Most emphasized the importance of family support. Before such a self-designed dietary program, none had consulted a dietitian, and only a few had received brief nutritional advice from their physician. Nearly all (*n* = 9) reported physical inactivity due to time constraints or a lack of suitable facilities, and most (*n* = 8) slept 5–6 h per night. The majority did not believe remission was possible, although two remained hopeful.

Finally, the group of dietitians (*n* = 10) noted that most patients with T2DM do not seek dietary care unless referred by a physician. Such referrals were more common in medical centers but remained generally infrequent overall. Adherence rates reached 70–90% when patients were referred by their physician, compared to 40% without referral, and remission was often observed with strict adherence to dietary treatment. Standard dietary prescriptions emphasized high fiber intake, low sugar, low fat, moderate complex carbohydrates, and foods with a low glycemic index. When asked about barriers that may hinder adherence in patients with T2DM, dietitians identified many, such as illiteracy, financial hardship, restrictive diets, lack of support, and time constraints. To improve adherence, they recommended flexible meal plans, ongoing encouragement, empathetic counseling, and family member involvement in the care process.

Based on the needs assessment and informed by the focus group findings, the problem’s logic model was developed and presented in Table 1. This logic model outlines how personal and environmental determinants influence treatment adherence among patients with T2DM and, in turn, impact health and quality of life.

**Table 1:** Logical model of the problem based on the PRECEDE model (101)—weight loss and remission of T2DM in Northern Lebanon.

Phase	Level	Components	Description
Phase 1: Quality of life outcomes	Quality of Life Outcomes	- Increased health expenses - Poor quality of life - Increased stress	Overall impact on patients’ well-being
Phase 2: Health outcomes	Health Outcomes	- Increased complications of T2DM - Decreased likelihood of diabetes remission	Clinical consequences of inadequate management
Phase 3: Behavioral and environmental conditions	Behavioral & Environmental Conditions	- Unhealthy lifestyle - High stress levels - Limited health literacy - Low confidence in self-care - Limited access to dietitians - Budgetary constraints	Factors directly influencing disease management
Phase 4: Determinants (individual) Determinants (environmental)	Individual Determinants	- Limited knowledge of diabetes and nutrition - Low perceived behavioral control - Poor medication adherence - Limited planning skills - Physical inactivity - Insufficient sleep duration - Poor stress coping - Low motivation	Personal-level determinants
	Environmental Determinants	- Limited family support - Limited financial resources - Limited access to healthcare services - Limited referral to dietitians	External-level determinants

Based on the logic model of the problem and the findings from the needs assessment, we formulated program goals that clarify the purpose of our clinical intervention.

We have established four goals:

1. Promote dietary adherence and self-management behaviors among patients with T2DM in North Lebanon through structured, culturally appropriate nutrition counseling.
2. Achieve sustainable weight loss and improved glycemic control in patients with T2DM, to reduce diabetes-related complications and support potential remission
3. Enhance patient knowledge, self-efficacy, and motivation regarding diabetes management through individualized education and regular follow-up.
4. Reduce the socioeconomic burden of poorly controlled diabetes by promoting cost-effective, non-pharmacological interventions focused on lifestyle changes.

### Step 2: logic model of change

The second step of the IM protocol centers on identifying target changes at both the behavioral and environmental levels.

To achieve the program goals, specific behavioral outcomes (BOs) that participants need to adopt for successful intervention implementation have been identified. These outcomes transform the program’s broad aims into measurable, observable behaviors that directly contribute to each goal identified in the previous step.

To make our planning more straightforward, we have grouped these outcomes into five domains that focus on related behaviors. These domains include “dietary adherence and eating behavior,” “weight loss and glycemic control,” “PA and lifestyle,” “adherence to medical care and support system,” and “relapse prevention and maintenance.” Each BO represents a modifiable behavior that positively impacts glycemic control, weight loss, or long-term adherence in individuals with T2DM (52, 65). This structured approach aligns with IM principles for creating effective logic models of change (54). The BOs are listed in Box 1.

**BOX 1 Behavioral outcomes.**

**Domain 1: Dietary adherence & eating behavior**

1 Adhere to the prescribed dietary plan (LC or MD) by establishing regular meal patterns, controlling portion sizes, and improving the quality of food choices to support diabetes remission.
2 Prepare meals aligned with the prescribed diet.

**Domain 2: Weight loss & glycemic control**

3 Achieve and maintain 10–15% weight loss to support blood glucose control and reduce metabolic complications (dyslipidemia and low-grade inflammation).

**Domain 3: Physical activity (PA) & lifestyle**

4 Engage in at least 150 min of PA per week.
5 Manage stress to support dietary adherence, lifestyle choices, and emotional well-being.
6 Get adequate sleep to support metabolic and behavioral regulation.

**Domain 4: Adherence to medical care & support systems**

7 Adhere to prescribed oral anti-diabetic medications.
8 Attend scheduled monthly follow-up sessions throughout the intervention.
9 Utilize the mobile app to sustain new behaviors and support adherence during the maintenance phase (phase 2 of the intervention).

**Domain 5: Relapse prevention & maintenance**

10 Maintain diet and lifestyle changes during the maintenance phase for continuous blood glucose control.
11 Respond effectively to relapse and return to target behaviors.

These BOs were selected based on insights drawn from literature research on diabetes self-management (66, 67) and various behavior change theories used for diabetes management and weight loss (68–70).

In addition to the BOs, several environmental factors can influence behavior change; in this intervention, we specifically address family support as a key determinant, based on findings from focus group discussions that underscore its importance for adherence to dietary and lifestyle changes.

After identifying the BOs, the next step is to set specific, observable Performance Objectives (POs), outlined in Supplementary material S1, to achieve the overall behavioral goals. By breaking down each BO into distinct actions, we can effectively focus our intervention strategies, identify what drives change, and determine the most effective methods to achieve it. This organized approach creates a clear pathway from the desired behaviors to the content of the intervention, following the guidelines of the IM protocol (54).

Having identified the POs, the next step is to select the key determinants that influence these behaviors to create Change Objectives (COs). These COs will guide the choice of theory-based methods and practical strategies in the intervention.

The determinants chosen for developing COs—knowledge, self-efficacy/skills, and attitude- were based on both theory and empirical evidence. These determinants are well-established predictors of health behavior change and are frequently used in the management of T2DM and among individuals who are overweight (71–73). Knowledge, a component of behavioral capability in social cognitive theory (SCT), was included to ensure that participants are well-equipped with sufficient information regarding weight loss, weight maintenance, and diabetes control, since health illiteracy was one of the critical barriers impeding treatment adherence, as mentioned earlier in step 1 (74, 75). Self-efficacy, the confidence to perform a specific behavior despite obstacles, and skills, the practical capabilities required to carry out specific self-care tasks, were both incorporated due to their strong association with sustained behavior change and dietary self-regulation (76) and were both supported by “diabetes self-management education” (77). Addressing these determinants will help patients maintain the changes made during treatment, especially as they encounter challenges with medication adherence and weight-loss strategies, as previously mentioned. As for attitude, valuing the required change as beneficial and motivating is crucial for sustaining new behaviors (73). The chosen determinants also align with the American Diabetes Association’s framework for practical diabetes self-management education and support, as well as recommendations for weight loss (78–80), due to their high relevance and adaptability.

Following the identification of relevant determinants, the next task was to integrate them with POs to develop a comprehensive matrix of change outlining the specific changes needed at the individual level to facilitate behavior change. For instance, participants must not only understand how losing weight can aid in diabetes remission, but they must also believe in the benefits of adhering to a diet and feel capable of overcoming any obstacles that may arise.

This approach ensures that the intervention addresses not only what participants need to do but also why they may struggle to do it and how to support them effectively. An example of “matrix of change for BO2” including the POs and COs is presented in Table 2, whereas the entire matrices for all BOs are listed in the Supplementary material S1.

**Table 2:** Matrix of change objectives for BO2.

BO2: Prepare meals aligned with the prescribed diet			
Performance objectives (POs)	Determinants		
	Knowledge	Skills/ Self-efficacy	Attitude
PO 1: List the necessary steps to prepare a balanced diet aligned with the dietary guidelines.	K1: Understand the dietary guidelines. K2: Understand the components of a balanced diet. K3: List the steps involved in meal planning.	S1: Be able to identify and select food that aligns with dietary guidelines SE 2: Feel confident in organizing balanced and culturally appropriate meals.	A1: Value the long-term benefits of well-planned meals on health and diabetes control.
PO 2: Develop a sample meal plan for two days	K1: Know how to distribute meals and snacks throughout the day. K2: Know how to use dietary guidelines to structure the meals (MD or LC).		A1: Believe that meal planning is achievable and worth the time and effort.

### Step 3: program design

The third step of the IM protocol involves identifying theory-based methods that influence determinants and translating them into practical strategies aligned with the COs (54).

After several meetings within the planning team and drawing on insights into the needs assessment, and guided by the matrices of change, the intervention was designed to include the following components:

1. Individual counseling sessions – focused on diet modification, weight loss strategies, diabetes education, and stress reduction.
2. PA promotion – guidance to initiate and sustain moderate PA.
3. Stress management and coping skills – practical techniques to reduce emotional eating and improve mental well-being.
4. Family support and social reinforcement – encouraging the involvement of family members to strengthen adherence.
5. Self-monitoring and feedback – use of tools (e.g., food logs, weight records) to track progress and reinforce efforts in the weight loss phase (phase 1), and the use of the mobile app in the maintenance phase (phase 2) to sustain the new behaviors.
6. Relapse prevention and problem-solving – preparing participants to anticipate setbacks and implement recovery strategies.

These intervention components included theory-based behavior “change methods” to address key factors such as knowledge, skills, self-efficacy, and attitudes. Our primary framework was SCT, which emphasizes self-efficacy, observational learning, and behavioral capability (76), essential aspects for dietary adherence and self-management in individuals with T2DM (81). We integrated practical strategies from SCT, such as guided practice, skills training, coping techniques, and modeling, into the program. SCT served as the leading theory addressing all determinants to increase knowledge, promote self-efficacy, enhance skills, and shape attitudes (82).

To complement the SCT, the Health Belief Model (HBM) was applied to ensure that health information was relevant and motivational (83). It guided the intervention in shaping patients’ attitudes toward meal planning and dietary adherence by emphasizing risk perception and personal responsibility (84). Both SCT and HBM were adopted in a study done to improve the self-management of diabetes in Alabama (85).

We also employed Cognitive Learning Theory (CLT) (86) to improve the understanding of knowledge areas, ensuring that new information was presented in a manner consistent with learners’ cognitive abilities (87). In the “Knowledge” section, this theory was employed to highlight the significance of weight loss in achieving diabetes remission, equipping participants with the necessary knowledge and motivation to initiate lifestyle changes and become physically active.

Finally, we incorporated the Transtheoretical Model (TTM) (88) to assess and enhance participants’ readiness for change, guiding them through the stages from awareness to sustained action (89). It was used to strengthen knowledge through consciousness-raising and attitudes through self-evaluation within the intervention.

After selecting the most relevant theoretical methods, we operationalized them into practical applications tailored to our target population. Table 3 presents an example of the resulting matrix, linking one PO to its corresponding COs, determinants, theory-based methods, and specific strategies. The whole table for all BOs is available in Supplementary material S2.

**Table 3:** Theory-based method and practical strategies for BO2.

BO2: Express confidence and skills in preparing meals aligned with the prescribed diet			
	Change objective	Theoretical method-relevant theory	Practical strategy
PO 1: List the necessary steps to prepare a balanced diet aligned with the dietary guidelines.	K1: Understand the dietary guidelines.	Didactic instruction (SCT). Active information processing (CLT). Feedback (SCT).	Inform participants about the dietary guidelines that define a healthy, balanced diet.
	K2: Understand the components of a balanced diet.		Ask the participant to list the components of the previously introduced balanced diet. Provide immediate feedback.
	K3: List the steps involved in meal planning.		The participant is asked to list the steps involved in meal planning. The participant should provide an example of meals composed based on the principles taught. Immediate feedback is provided.
	S1: Be able to identify and select food that aligns with dietary guidelines.	Skills training (SCT). Feedback (SCT).	The dietitian shows the participant many food items and asks him to identify which item fits his dietary patterns. Provide him with immediate feedback.
	SE 2: Feel confident in organizing balanced and culturally appropriate meals.		The dietitian asks the participant to prepare three balanced, complete meals that fit his cultural background. Provide him with immediate feedback.
	A1: Value the long-term benefits of well-planned meals on health and diabetes control	Reinforcement Through Direct Experience (SCT)	Show participants the benefits of choosing balanced meals, including weight and fat loss, improved blood glucose control, and an overall more optimal metabolic profile. For this purpose, at each clinical visit, the participant’s body composition will be assessed using bioelectrical impedance, and his blood will be tested every three months.

Referring to Table 3, to address PO1, for example, along with its related objectives in knowledge (K), self-efficacy (SE), and skills (S), the patient is first educated about dietary guidelines for a healthy meal (K1, K2) through didactic instruction and active information processing. These approaches ensure that the patient can accurately identify the nutritional components and steps involved in meal planning. To enhance skills (S1) and build self-efficacy (SE2), tangible examples are provided, followed by guided practice in selecting and organizing culturally appropriate meals. Finally, in alignment with attitude A1 “Value the long-term benefits of well-planned meals,” reinforcement through direct experience- such as demonstrating improvements in body composition and blood glucose levels- helps to maintain motivation, consistent with the principles of SCT.

### Step 4: program production

In the fourth step of IM, the previously identified theoretical methods and strategies were translated into practical, concrete, and user-friendly intervention materials. Following a series of virtual and face-to-face collaborative meetings, a planning team of six dietitians finalized a comprehensive counseling manual to guide standardized delivery of both dietary interventions. These dietitians from diverse cultural backgrounds met to discuss the characteristics of patients living in their respective regions, specifically in the North of Lebanon, as these traits can vary by religion, educational level, and socio-economic status.

Based on the findings of the needs assessment and the defined program objectives, the team developed all intervention materials, including structured questionnaires, dietary plans, tracking sheets, and instructional handouts. The materials provided included an introduction to the trial, highlighting the role of the low-calorie MD and low-calorie LC diets in achieving remission from T2DM, and dietary guidance for each dietary approach, including food lists, sample meal plans, and portion-control guides.

To ensure clarity, cultural relevance, and acceptability, all materials were written in Arabic and pilot-tested with a small sample of target participants (*N* = 6). During this pilot testing, dietitians administered the questionnaires while documenting completion time, item clarity, and any misunderstandings. Revisions were made to improve readability and contextual appropriateness. For instance, additional questions were incorporated to assess daytime sleep hours, recognizing the cultural norm of afternoon napping. Meal plans were designed to be affordable, given Lebanon’s ongoing economic crisis. Religious considerations were respected; alcohol was excluded for Muslim participants, and affordable alternatives to fatty fish, such as canned tuna, canola oil, and almonds, were included to maintain adequate omega-3 intake, a key component in the MD.

To support intervention fidelity, dietary intake will be assessed at each consultation using a 24-h dietary recall. When necessary, participants will be asked to complete a multiple-day food record to allow for a more detailed evaluation of dietary adherence. For participants assigned to the MD arm, adherence will additionally be assessed at each visit using the Mediterranean Diet Adherence Screener (MEDAS) questionnaire (90). All participants will be seen individually to minimize confusion between dietary prescriptions and to reduce the risk of contamination between intervention arms.

For the second phase of the trial (maintenance phase), a visual communication tool, a mobile application, was developed to help participants sustain the new behaviors learned in phase one and maintain their weight. The app included features for tracking anthropometric measurements, blood glucose, blood pressure, diet adherence, and PA, as well as a private chat room for direct communication with dietitians. A detailed description of the app’s development process will be published separately. Before the start of the second phase, a subsample of 10 participants will test the mobile app to evaluate its usability.

Throughout the trial (12 months in total), participants continued regular monthly follow-up visits with dietitians, and blood tests were conducted every 3 months. All participants provided informed consent before enrollment.

### Step 5: creation of an adoption and implementation plan

In the fifth step of IM, the planners have four tasks to accomplish:

- Identify potential program users
- State outcomes and POs for program use
- Construct matrices of COs for program use
- Design implementation interventions

#### Task 1: identify potential program users

To support real-world applications, the program was designed to fit within routine clinical care in primary health settings. The adoption of the program will rely on the willingness of medical center directors and private physicians to approve its integration into regular services, by promoting dietary referrals and providing logistical support, such as staff time and counseling space. Furthermore, private dietetic clinics will be utilized to implement the program, with dietitians from the research team serving as the respective practitioners.

At the adoption level, we expect primary medical center directors to agree to incorporate the program into their existing workflows and encourage their staff to participate, as we anticipate better referrals from physicians for dietary consultations.

At the implementation level, we aim for dietitians to deliver the intervention consistently and in line with the protocol, using culturally appropriate tools and strategies.

#### Task 2: state outcomes and POs for program use

This task outlines the expected behaviors of key users- dietitians, physicians, and directors of primary medical centers- at the levels of adoption, implementation, and maintenance, while preserving fidelity to the intervention design (54).

##### Adoption outcomes include

Directors of primary medical centers approve the integration of the program into routine care and allocate time and space for the dietitian to conduct the dietary assessment at each patient’s visit.

Physicians refer patients with T2DM to participating dietitians.

- Dietitians express positive attitudes toward the feasibility and usefulness of the program and its materials.
- Dietitians commit to adopting the program in clinical practice and participating in related preparatory training.

##### Implementation outcomes include

Dietitians are expected to:

- Attend training sessions and demonstrate understanding of the intervention protocol.
- Deliver the intervention through structured, multiple counseling sessions while maintaining fidelity to the protocol’s core components.
- Monitor and engage patients throughout the intervention, providing motivation and support to encourage adherence to dietary and behavioral recommendations.

##### Maintenance outcomes include

- Directors and physicians express satisfaction with the program’s feasibility and clinical outcomes.
- Dietitians report confidence in continuing to apply the program’s components after the trial.
- Directors of dietetic clinics and medical centers demonstrate interest in sustaining or institutionalizing the program as part of routine care.

##### Performance objectives (POs)

###### PO for dietitians

- PO1: Attend and actively participate in the training sessions to prepare for implementation.
- PO2: Plan and hold counseling sessions in accordance with the study protocol.
- PO3: Use tracking tools and mobile app consistently to monitor patient progress throughout the study.

###### PO for adopters (clinic directors and physicians)

- PO1: Physicians refer eligible patients to trained dietitians.
- PO2: Dietitians communicate with referring physicians when needed for patient care coordination.
- PO3: Clinic directors allocate necessary resources (e.g., time, space, staff support) to support the implementation of the counseling sessions.

#### Task 3: construct matrices of change objectives for program use

Matrices of COs were systematically developed to link each PO with its corresponding behavioral determinants, including knowledge, self-efficacy/skills, perceived feasibility, and attitude, for both dietitians and program adopters (physicians and clinic directors). These matrices delineate the specific behavioral and cognitive changes required to achieve the intended adoption, implementation, and maintenance outcomes.

A comprehensive presentation of the matrices, detailing the relationships between POs and their associated determinants, is provided in Supplementary material S3.

#### Task 4: design implementation interventions

To facilitate effective integration of the intervention into routine practice, specific implementation strategies were developed based on the identified determinants and guided by the SCT and HBM. All strategies were designed by the planning team to fit the operational realities of primary healthcare in the Middle East, ensuring feasibility, cultural appropriateness, and sustainability beyond the trial period.

##### For dietitians

Interactive workshops incorporating modeling, guided practice, and feedback build skills and self-efficacy in using the counseling manual, tracking tools, and mobile app. Role-playing with culturally relevant scenarios reinforces confidence and motivation (SCT constructs: mastery experience, modeling, reinforcement).

##### For physicians

Brief informational sessions highlight the benefits of dietary interventions for diabetes remission and provide a simple referral protocol to facilitate patient access to dietetic care. These sessions aim to increase perceived benefits and reduce perceived barriers to referral (HBM constructs: perceived benefits and perceived barriers).

##### For medical center directors

Individual meetings present the program structure, anticipated outcomes, and resource needs. These meetings aim to foster organizational commitment, reduce structural barriers, and enhance readiness for adoption (HBM construct: enabling conditions).

#### Step 6: planning for evaluation

This final step in the IM process focuses on developing a plan to evaluate the effectiveness and implementation of the intervention (54). While this manuscript centers on the design of the first phase of the trial using the IM approach, preliminary evaluation plans have been developed to ensure a comprehensive assessment in subsequent stages. The trial outcomes will be evaluated to determine the intervention’s effectiveness in achieving diabetes remission across two diets, and to assess the impact of both the MD and LC diets on weight loss, glycemic control, and diabetes-related metabolic complications. Additionally, the evaluation will assess the feasibility and fidelity of the implementation. It will examine multiple dimensions, including the quality of communication between patients and dietitians, adherence to behavioral changes, and assigned dietary interventions.

On the other hand, the maintenance phase (Phase 2) will include an evaluation of the mobile application’s role in supporting adherence and facilitating communication. Implementation outcomes will also be assessed, including program fidelity, participant acceptability of the diets, and the appropriateness of the tracking tools and questionnaires. These evaluation components will provide valuable insights into both the intervention’s effectiveness and its feasibility for integration into routine clinical practice. Further details will be presented in subsequent articles concerning the mobile app design and features, as well as the intervention results.

## Discussion

This article describes the systematic planning and development of a two-arm dietary intervention for patients with T2DM in Lebanon, guided by the IM protocol. The intervention aims to achieve diabetes remission through weight loss and improve diabetes outcomes among patients with T2DM in Lebanon. The IM framework integrates evidence-based behavior change techniques that support motivation, self-monitoring, habit formation, self-efficacy, and social support, while also addressing both physical and psychological resources (54, 91). This approach has been successfully applied in previous health promotion programs targeting weight loss and diabetes self-management (92, 93), as it addresses key determinants of obesity, a primary risk factor for T2DM, and supports long-term lifestyle changes (36).

The intervention targets overweight adults with T2DM living in Northern Lebanon, a region marked by high rates of poverty, limited access to healthcare services, and an overburdened infrastructure due to a large refugee population comprising approximately 28% of the area’s residents (94). These contextual factors, combined with a documented gap in diabetes knowledge and poor attitudes toward disease management among Lebanese patients [78], highlight the urgent need for a tailored, accessible intervention. To reach this vulnerable population, the intervention will be implemented in both private clinics and primary medical care centers, the latter being the main healthcare access point for underserved communities. These primary care centers offer free medical consultations and anti-diabetic medications, often supported by foreign aid, making them ideal settings for delivering cost-effective interventions. Implementing the intervention program within existing healthcare structures enhances its feasibility, adoption, and sustainability in resource-limited settings. The intervention includes culturally adapted low-calorie dietary plans, either the MD or LC diet, designed to be affordable and acceptable within the local context. Evidence consistently shows that culturally tailored interventions improve participant engagement, adherence, and clinical outcomes in diabetes management compared to generic programs (95). Participants will receive continued support from trained dietitians through in-person counseling, WhatsApp check-ins, and a custom-built mobile app to enhance adherence and maintain weight loss over time. Combining face-to-face and virtual education allows for continuous guidance and flexibility, helping participants stay engaged despite geographic and socioeconomic barriers (96). Evidence shows that tele-education can be as effective as in-person education in improving diabetes education (96, 97). This hybrid approach ensures accessibility, continuity of care, and sustained motivation throughout the intervention.

## Strengths and limitations

To date, most interventions addressing weight loss and diabetes remission have relied on very low-calorie diets using specialized formula products (18, 29, 98, 99). To our knowledge, no published trials in Lebanon have specifically evaluated comprehensive dietary interventions aimed at achieving diabetes remission. This intervention adopts a more culturally appropriate and sustainable approach by promoting the consumption of everyday home-prepared foods. This strategy enhances the program’s feasibility and long-term applicability within the local context. Moreover, the dietary plans are individualized to account for each patient’s medical history, cultural background, and economic situation, increasing the likelihood of meaningful and realistic outcomes.

Nonetheless, challenges are anticipated. A substantial portion of the target population has low levels of education and socioeconomic status, which may hinder adherence to dietary recommendations and regular participation in counseling sessions (57, 58, 100). Additionally, the current referral system is not well established, as many physicians- particularly endocrinologists- tend to provide dietary advice themselves without referring patients to dietitians. One of the intended long-term outcomes of this intervention is to foster a more collaborative, multidisciplinary care model that encourages appropriate referrals to nutrition professionals. Finally, the trial is conducted in Northern Lebanon and may not be applicable to other regions due to differences in dietary habits, cultural practices, and healthcare systems. The selection of participants based on specific criteria may limit the generalizability of the findings, particularly for individuals with advanced diabetes or varying treatment and sociodemographic profiles. To establish broader external validity, future studies should include more diverse populations and healthcare contexts.

In the present intervention, these challenges can be addressed by encouraging primary medical centers to adopt the program, as these centers provide free or low-cost consultations and facilitate internal referrals among health practitioners, including dietitians. Moreover, primary medical centers are widely distributed across multiple villages and neighborhoods, allowing patients to access care close to home. This proximity reduces transportation barriers, promotes continuity of care, and enables patients to remain within their cultural and social environment while receiving a culturally tailored intervention. Such integration into familiar community settings can enhance participation, adherence, and the program’s overall effectiveness.

## Conclusion

This two-phase intervention, planned to follow the IM protocol steps, aims to build a supportive system for individuals in Northern Lebanon who are struggling with weight loss and diabetes management, within the context of limited healthcare resources. By offering a culturally relevant, theory-informed intervention that combines low-calorie MD and low-calorie LC diets, the program aims to help participants achieve meaningful improvements in their health, enabling them to lead a higher-quality life.

We acknowledge the significant challenges faced by many in this region, including limited access to care, economic hardship, and low health literacy. After the intervention is implemented, we will evaluate its effectiveness, assess its implementation, and explore its potential for adaptation in other low-resource settings. Through this work, we aim to contribute to the global response to T2DM by promoting practical, accessible, and empowering strategies for healthier living.
