# Supplementary material for: A randomized controlled trial protocol comparing low-calorie Mediterranean and low-carbohydrate diets for diabetes remission in individuals with type 2 diabetes in northern Lebanon: an intervention mapping–based approach
Source: Front Public Health. 2026 Apr 8;14:1787980. doi: 10.3389/fpubh.2026.1787980 (PMC13100829; doi:10.3389/fpubh.2026.1787980)
Supplement: Supplementary file 1 [file Supplementary_file_1.docx]

**Supplementary material- S 1 – Table 2**

**Step 2 of IM**

**Complete matrix of change objectives**

| BO1. Adhere to the prescribed dietary plan (LC or MD) by establishing regular meal patterns, controlling portion sizes, and improving the quality of food choices to support diabetes remission | | | |
| --- | --- | --- | --- |
| Performance objectives (POs) | **Determinants** | | |
|  | **Knowledge (K)** | **Self-efficacy/Skills (SE/S)** | **Attitude (A)** |
| PO1: Follow a structured dietary plan to support weight loss, improve glycemic control, and reach diabetes remission. | K1: Know the causal link between being overweight and T2DM.  K2: Recognize the importance of weight loss for diabetes remission.  K3: Understand the beneficial effects of diabetes remission on health, step-down therapy, and socioeconomic status.  K4: List at least three essential steps to achieve diabetes remission (weight loss, lifestyle changes) | SE 1: Feel confident to stick to the prescribed dietary pattern.  SE 2: Feel able to overcome obstacles and achieve diabetes remission. | A1: View dietary and lifestyle changes as an investment in long-term health.  A2: Believe that small changes in food habits can lead to significant results.  A3: Be optimistic about the possibility of remission through personal efforts. |
| PO2: Eat three main meals per day following the MD or LC diet | K1: Understand the benefits of regular meal timing (blood glucose control, energy balance, and appetite regulation).  K2: List three benefits of the selected diet.  K3: Recognize the risks of skipping meals. | S1: Know how to plan and prepare meals, even during busy times. | A1: Believe that eating regularly helps manage appetite and blood glucose.  A2: Believe that skipping meals will negatively affect health and trigger overeating.  A3: Value routine and believe it’s worth making time for meals. |
| PO3: Choose appropriate low-calorie snacks between main meals | K1: List five suitable snack options based on the chosen diet | S1: Be able to choose a healthy snack in different settings | A1: Value health over taste and convenience |
| PO4: Detect and solve challenges to regular meal consumption | K1: Know common challenges (time constraints, social pressure) and their solutions | SE 1: Feel confident in managing barriers | A1: Believe in and value the importance of overcoming those barriers |
| PO5: Monitor and track food intake regularly | K1: Understand how tracking supports mindful eating and weight management.  K2: Recognize how tracking improves awareness and control. | S1: Be able to respond to specific tools to recall food intake (24-hour recall, food record) | A1: Believe that tracking food intake is valuable and worth the effort. |
| PO 6: Practice portion control by selecting healthy serving sizes | K1: Know recommended portion sizes for different food groups | S 1: Know how to estimate portion sizes when eating | A1: Believe that knowing serving sizes helps control weight |
|  | K2: Know how to use the household items as a visual guide to estimate portions. | S 2: Be able to use visual cues or tools to guide portions | A2: Value portion awareness as a tool for control, not restriction |
|  | K3: Know that portions vary based on individual calorie requirements. | S 3: Know how to adjust the portion size based on the calorie needs, as indicated by the dietitian, and in response to variations in blood glucose levels. | A3: Believe that adapting portion size to personal needs is essential for achieving health goals. |
|  | K4: Understand how portion control affects hunger, satiety, and blood glucose. |  | A4: Believe that adjusting portions is a flexible, self-care routine. |
| PO7: Identify foods that align with recommended dietary patterns (MD or LC) | K1: Know which foods are considered healthy or unhealthy within the chosen diet. | SE 1: Feel confident in identifying and choosing compliant foods.  S 1: Be able to prepare meals from basic, whole ingredients | A1: Believe that knowing what to eat is essential to managing weight and blood glucose. |
| PO8: Prepare meals using whole, minimally processed ingredients | K1: Know which ingredients to prioritize |  |  |
| PO9: Read food labels to compare nutritional quality | K1: Know how to interpret dietary facts, especially total carbohydrate, for glucose control | S 1: Be able to use food labels to select healthier options. | A1: Believe that label reading is essential to managing health. |

| BO2: Prepare meals aligned with the prescribed diet | | | |
| --- | --- | --- | --- |
| Performance objectives (POs) | **Determinants** | | |
|  | **Knowledge** | **Skills/ Self-efficacy** | **Attitude** |
| PO 1: List the necessary steps to prepare a balanced diet aligned with the dietary guidelines. | K1: Understand the dietary guidelines.  K2: Understand the components of a balanced diet.  K3: List the steps involved in meal planning. | S1: Be able to identify and select food that aligns with dietary guidelines  SE 2: Feel confident in organizing balanced and culturally appropriate meals. | A1: Value the long-term benefits of well-planned meals on health and diabetes control. |
| PO 2: Develop a sample meal plan for two days | K1: Know how to distribute meals and snacks throughout the day.  K2: Know how to use dietary guidelines to structure the meals (MD or LC). |  | A1: Believe that meal planning is achievable and worth the time and effort. |
| BO3: Achieve and maintain 10-15% weight loss to support blood glucose control, diabetes remission, and reduce metabolic complications (dyslipidemia and low-grade inflammation). | | | |
| Performance objectives (POs) | **Determinants** | | |
|  | **Knowledge** | **Skills/ Self-efficacy** | **Attitude** |
| PO1: Follow the prescribed low-calorie diet for the assigned duration. | K1: Know the benefits of losing weight on health and blood glucose control. | S 1: Be able to integrate the low-calorie diet with physical activity while self-monitoring food intake for an appropriate weight loss. | A1: Believe that following good eating patterns is a long-term practice for better health |
| PO2: Be physically active for a minimum of 150 minutes per week. | K1: Understand the benefits of physical activity (PA) on weight loss and glucose control. |  | A1: Believe that regular physical activity is essential to losing weight. |
| BO4: Engage in at least 150 minutes of physical activity (PA) per week | | | |
| Performance objectives (POs) | **Determinants** | | |
|  | **Knowledge** | **Skills/ Self-efficacy** | **Attitude** |
| PO1: Set achievable and realistic goals for PA practice that fit with diabetes control | K1: Recognize the importance of regular physical activity (PA) in weight management and diabetes control.  K2: Be aware of the recommended guidelines for physical activity (PA) duration and intensity. | SE 1: Be able to set realistic and personalized goals. | A1: Value “goal setting” as a tool for controlling weight, PA, and health issues.  A2: Understand that setting small, realistic goals is more sustainable than drastic ones. |
| PO2: Incorporate PA in daily routine. | K1: Recognize the importance of PA in the daily routine to control appetite and blood sugar levels.  K2: Know how to adjust glycemia based on the intensity and duration of physical activity. | S1: Know how to coordinate the food intake with the type and intensity of exercise. | A1: Feel motivated to make active choices during the day (climbing stairs instead of taking the elevator…) |
| PO3: Monitor and address barriers to being active. | K1: Understand common barriers to PA.  K2: Know practical strategies to overcome the barriers.  K3: Understand that barriers are common and manageable. | SE 1: Feel confident in adapting and adjusting the goals when needed without giving up.  SE 2: Believe in their ability to follow the weekly plan despite a busy schedule.  SE 3: Ability to stay motivated and track progress regularly | A 1: Value the habit of self-reflection and problem-solving in maintaining the PA routine. |
| BO5: Manage stress to support dietary adherence and emotional well-being. | | | |
| Performance objectives (POs) | **Determinants** | | |
|  | **Knowledge** | **Skills/ Self-efficacy** | **Attitude** |
| PO1: Utilize a coping strategy when experiencing stress. | K1: Know the connection between stress and emotional eating.  K2: Know the common signs of stress.  K3: Know some evidence-based coping strategies (deep breath, PA, problem solving) | S1: Be able to recognize stress triggers and respond adequately | A1: Believe that stress management is essential for maintaining behavior change |
| PO2: Plan for PA to reduce stress | K1: Know that PA is an appropriate coping strategy to relieve stress by improving mood.  K2: Know how to integrate PA in stressful moments. | SE 1: Feel confident to engage in PA when feeling stressed. | A1: Believe that PA is a valid, effective, and sustainable way to relieve stress. |
| PO3: When uncontrolled emotional eating occurs, choose low-calorie items | K1: Know a list of low-calorie, self-satisfying items.  K2: Know the difference between physiological hunger and emotional hunger. | S1: Be able to select a low-calorie item in moments of stress.  S2: Be able to identify emotions and triggers before reaching for food. | A1: View this behavior as a step toward self-regulation. |
| BO6: Get adequate sleep to support metabolic and behavioral regulation. | | | |
| Performance objectives (POs) | **Determinants** | | |
|  | **Knowledge** | **Skills/ Self-efficacy** | **Attitude** |
| PO1: Plan to sleep for at least 8 hours/night. | K1: Know the importance of adequate sleep to control glycemia (allow normal hormonal fluctuations) and lose weight.  K2: Know the impact of sleep deprivation on craving and dietary decisions. | S1: Be able to set and stick to a regular bedtime.  S2: Be able to identify and manage barriers to sleep.  S3: Be able to use relaxation techniques before sleeping (ex, reading) | A1: Value sleep as an initial component for appropriate weight loss and blood glucose control.  A2: Value the importance of sleep for optimal physical and mental health. |
| PO2: Follow a healthy lifestyle, like being physically active and decreasing caffeine intake, to improve sleeping quality. | K1: Know evidence-based tips that improve sleep quality, such as regular physical activity, caffeine reduction, and consistent sleeping routines. |  |  |
| BO7: Adhere to prescribed oral anti diabetic medications | | | |
| Performance objectives (POs) | **Determinants** | | |
|  | **Knowledge** | **Skills/ Self-efficacy** | **Attitude** |
| Set an alarm or a specific time to take the medication. | K1: Be aware of the importance of taking the medication consistently to prevent complications.  K2: Understand the effect of weight loss on step-down therapy (reduction of oral antidiabetic medication) | SE 1: Be able to stick to the medication regimen and adjust food intake accordingly. | A1: Value the role of medication to control glycemia alongside diet and PA.  A2: Believe in the importance of losing weight on step-down therapy and the resultant health and economic benefits. |
| BO8: Attend scheduled monthly follow-up dietetic sessions throughout the intervention. | | | |
| Performance objectives (POs) | **Determinants** | | |
|  | **Knowledge** | **Skills/ Self-efficacy** | **Attitude** |
| PO1: Schedule sessions in advance. | K1: Understand the importance of consistent follow-up for managing dietary intake and overall health. | SE 1: Feel capable of fitting appointments into the personal schedule. | A1: Value proactive planning to maintain consistent care. |
| PO2: Actively participate in the sessions. | K1: Understand the benefits of active engagement (asking questions, sharing progress, and challenges). | SE 1: Feel confident in sharing your concerns with the dietitian. | A1: Value the follow-up as beneficial and empowering. |
| PO3: Reschedule for missed sessions. | K1: Understand that missing a session is not a failure; rather, it is essential to reschedule and stay on track. | S1: Feel able to manage unexpected barriers. | A1: Rescheduling as a responsive behavior. |
| BO9: Utilize the mobile app to support adherence during the maintenance phase. | | | |
| Performance objectives (POs) | **Determinants** | | |
|  | **Knowledge** | **Skills/ Self-efficacy** | **Attitude** |
| PO1: Review progress reports, feedback, or reminders generated by the app | K1: Learn how to use the app interface effectively.  K2: Understand the purpose of logging data (monitor progress, receive feedback, identify patterns).  K3: Know the importance of maintaining weight loss. | SE 1: Believe that consistency in using the app supports long-term maintenance.  S1: Be able to interpret app-generated data (progress reports, feedback, reminders) and use it to guide decisions about behavior. | A1: Value the role of self-monitoring in maintaining weight loss.  A2: Feel motivated to engage with the app regularly. |

| BO10: Maintain diet and lifestyle changes during the maintenance phase for continuous blood glucose control. | | | |
| --- | --- | --- | --- |
| Performance objectives (POs) | **Determinants** | | |
|  | **Knowledge** | **Skills/ Self-efficacy** | **Attitude** |
| PO1: Continue with regular PA for a minimum of 150 minutes/week | K1: Know the importance of PA in weight maintenance and glycemia regulation | SE 1: Feel confident to plan and maintain PA even in a busy schedule | A1: Value the PA as an essential component to support weight maintenance |
| PO2: Continue to monitor weight at home. | K1: Know how to measure the weight accurately. | S1: Be able to initiate corrective action when weight is regained | A1: Value weight monitoring as a self-care and empowering practice that provides control over one's health. |
| PO3: Continue with monthly follow-up with the dietitian via face-to-face interview or through the app | K1: Understand the benefits of regular follow-up for maintaining weight and controlling blood glucose levels. | SE1: Feel capable of booking a monthly session or using the app. | A1: Value monthly follow-up as a part of diabetes management |
| PO4: Check blood glucose regularly and respond to essential fluctuations | K1: Recognize the symptoms of hypo- and hyperglycemia.  K2: Learn how to correct acute fluctuations, including hypoglycemia and hyperglycemia.  K3: Know the target blood glucose ranges.  K4: Know how to interpret blood glucose values and when to seek help. | S1: Be able to correct blood glucose fluctuations should they occur. | A1: Be motivated to use blood glucose data to make informed decisions about diet, PA, and medication. |
| PO5: Seek and utilize family support and cost-effective strategies to sustain dietary and lifestyle changes during the maintenance phase. | K1: Understand how family involvement and support can improve adherence to dietary and lifestyle changes.  K2: Identify affordable and healthy food options, as well as cost-saving practices. | SE 1: Feel confident in communicating needs and involving family in meal preparation if needed.  SE2: Believe in the ability to maintain a healthy diet on a budget. | A1: Value the role of family support and smart budgeting in maintaining long term health. |

| BO11: Respond effectively to relapse and return to target behaviors. | | | |
| --- | --- | --- | --- |
| Performance objectives (POs) | **Determinants** | | |
|  | **Knowledge** | **Skills/ Self-efficacy** | **Attitude** |
| PO1: Identify high-risk situations that may lead to relapse. | K1: Understand common triggers and high-risk situations that can lead to relapse.  K2: Understand strategies to prevent relapses (problem-solving planning, social support). | SE 1: Feel confident in the ability to recover and resume healthy habits. | A1: Value self-compassion and resilience over guilt. |
| PO2: Implement effective strategies when faced with a high-risk situation | K1: Understand different types of coping strategies (problem-focused versus emotion-focused).  K2: Be aware of harmful coping strategies like smoking. | SE1: Be able to maintain coping strategies in different situations. | A1: Value self-care and emotional regulation. |
| PO3: Monitor weight and dietary behaviors to prevent relapses. | K1: Recognize the importance of self-monitoring to avoid relapses and efficiently manage setbacks. | SE1: Feel confident in tracking food intake and weight consistently | A1: Believe that self-monitoring improves dietary habits and health. |
