# Supplementary material for: A randomized controlled trial protocol comparing low-calorie Mediterranean and low-carbohydrate diets for diabetes remission in individuals with type 2 diabetes in northern Lebanon: an intervention mapping–based approach
Source: Front Public Health. 2026 Apr 8;14:1787980. doi: 10.3389/fpubh.2026.1787980 (PMC13100829; doi:10.3389/fpubh.2026.1787980)
Supplement: Supplementary file 2 [file Supplementary_file_2.docx]

**Supplementary material- S 2- Table 3**

**Step 3 of IM**

**Theory-based method and practical strategies**

| BO1. Adhere to the prescribed dietary plan (LC or MD) by establishing regular meal patterns, controlling portion sizes, and improving the quality of food choices to support diabetes remission | | | |
| --- | --- | --- | --- |
|  | **Change objective** | **Theoretical method- Relevant theory** | **Practical strategy** |
| PO1: Follow a structured dietary plan to support weight loss, improve glycemic control, and reach diabetes remission. | K1: Know the causal link between being overweight and T2DM. | Active information processing (CLT)  Didactic instruction (SCT, HBM).  Consciousness Raising (TTM) | Structured delivery of information related to the effect of being overweight on T2DM, the beneficial effect of weight loss on glycemic control, step-down therapy, and potential diabetes remission.  Mention the beneficial effect on the reduction of medical expenses and the amelioration of the quality of life.  The information delivered is participant-centered, credible, and relevant.  All information is delivered by the research team, which is composed of dietitians. |
|  | K2: Recognize the importance of weight loss for diabetes remission. |  |  |
|  | K3: Understand the beneficial effects of diabetes remission on health, step-down therapy, and socioeconomic status. |  |  |
|  | K4: List at least three essential steps to achieve diabetes remission (weight loss, lifestyle changes) | Active information processing (CLT).  Feedback (SCT) | Provide the participant with clear and straightforward instructions on the steps required for diabetes remission.  Provide immediate feedback on whether the steps are correctly listed. |
|  | SE 1: Feel confident to stick to the prescribed dietary pattern.  SE 2: Feel able to overcome obstacles and achieve diabetes remission. | Self-monitoring (SCT).  Goal Setting (SCT).  Feedback (SCT)  Instructional therapy (SCT). | Tracking progress to increase awareness and belief in the ability to change, stick to the diet, and overcome challenges.  Setting achievable goals to build confidence in losing weight and achieve  diabetes remission.  The dietitian tracks food intake, weight loss, glycemia, and provides tailored feedback for progress.  *A 24-hour recall will be used at each visit to track food intake, and the MEDAS questionnaire will be used to assess adherence to the MD.* |
|  | A1: View dietary and lifestyle changes as an investment in long-term health. | Framing (HBM, prospective theory) | Emphasize the long-term rewards of dietary and lifestyle changes on health. |
|  | A2: Believe that small changes in food habits can lead to significant results. | Belief selection  (HBM) | Emphasize the importance of specific beliefs that small changes can lead to sustained glycemic and metabolic control. |
|  | A3: Be optimistic about the possibility of remission through personal efforts. | Value clarification (HBM).  Persuasive communication (SCT). | Engage participants in reflecting on how improved diabetes and its complications support their personal goals.  Target existing beliefs that “remission is not possible” and increase perceived benefits in both the short and long term, while encouraging lifestyle changes. |
| PO2: Eat three main meals per day following the MD or LC diet | K1: Understand the benefits of regular meal timing (blood glucose control, energy balance, and appetite regulation). | Consciousness raising (TTM). | Provide participants with both verbal and written information regarding regular meal timing and its impact on glycemia, appetite, and blood glucose levels. |
|  | K2: List three benefits of the selected diet | Active information processing (CLT).  Feedback (SCT). | Inform the participant verbally and write about the benefits of the selected diet (LC/MD).  Engage the participant to list the benefits of MD/LC on weight loss and diabetes.  Verify the accuracy of the listed benefits. |
|  | K3: Recognize the risks of skipping meals. | Consciousness raising (TTM). | Inform the participants about the potential problems that may arise if they do not follow the diet. |
|  | S1: Know how to plan and prepare meals, even during busy times | Instructional modeling/support  (SCT). | Engage participants in guided meal planning exercises using real-life schedules, where they select quick, balanced meals from provided recipes.  Provide the participants with easy and healthy recipes with household ingredients. |
|  | A1: Believe that eating regularly helps manage appetite and blood glucose. | Feedback  (SCT). | Track the participant’s food intake and correlate it with blood glucose measurements and appetite and provide him with feedback on this correlation. |
|  | A2: Believe that skipping meals will negatively affect health and trigger overeating. | Risk perception (HBM).  Feedback (SCT). | Showing a clear comparison between a person who skips meals and another who eats regularly.  Keep him updated on his blood glucose and blood lipid results and correlate them with his diet adherence.  *A blood test will be done at the beginning of the trial and every 3 months thereafter.* |
|  | A3: Value routine and believe it’s worth making time for meals. | Self- reevaluation  (TTM) | Show the participant that maintaining a healthy routine and eating regular meals can lead to lifelong benefits.  Benefits may include weight loss, fat loss, improved glycemic control, reduced blood lipid levels, and a better quality of life. |
| PO3: Choose appropriate low-calorie snacks between main meals | K1: List five suitable snack options based on the chosen diet | Didactic instruction (SCT)  Active information processing (CLT).  Feedback (SCT). | Introduce the concept of “healthy snacks” and provide examples of affordable, culturally relevant snacks.  Reinforce the knowledge by asking the participant to list five snack options suitable for the chosen diet (LC/MD).  Provide immediate feedback on whether the listed snacks are correct. |
|  | S1: Be able to choose a healthy snack in different settings | Skills training (SCT)  Feedback (SCT). | Participants should be able to choose from various snacks in different settings (e.g., at home, work, or while traveling).  Provide immediate feedback on whether the listed snacks are correct. |
|  | A1: Value health over taste and convenience | Value clarification (HBM).  Feedback (SCT) | The dietitian asks the participant to compare some healthy snacks to less healthy, convenient ones. The participant is asked to answer verbally, explaining the difference.  The dietitian provides immediate feedback on whether the answer is correct. |
| PO4: Detect and solve challenges to regular meal consumption | K1: Know common challenges (time constraints, social pressure) and their solutions | Didactic instruction (SCT).  Active information processing (CLT).  Feedback (SCT). | Introduce the challenges to regular meal consumption by giving examples on how to manage them.  Reinforce the knowledge by asking the participant to list some of the challenges he might face and mention their solutions.  Provide immediate feedback on whether the solutions are well chosen. |
|  | SE 1: Feel confident in managing barriers | Coping planning.  (SCT) | At each clinical visit, ask the participant about the barriers they faced and how they managed them. |
|  | A1: Believe in and value the importance of overcoming those barriers | Self-evaluation  (TTM) | During the clinical visit, the dietitian asks the participant whether they felt satisfied with overcoming the barrier and whether it was efficient and easy to do so. |
| PO5: Monitor and track food intake regularly | K1: Understand how tracking supports mindful eating and weight management | Didactic instruction (SCT).  Active information processing (CLT). | Explain to participants how tracking food intake helps them be aware of the quality of their food and control their eating habits to achieve weight loss.  Engage the participant in finding the gaps in his food intake. |
|  | K2: Recognize how tracking improves awareness and control. |  |  |
|  | S1: Be able to respond to specific tools to recall food intake (24-hour recall, food record) | Skills training (SCT).  guided practice (SCT). | Allow participants to try to recall food intake in a relaxed session with positive feedback.  A 24-hour recall is used at each clinical visit with the MEDAS questionnaire (for MD diet), and, if needed, a food record is also used. |
|  | A1: Believe that tracking food intake is valuable and worth the effort. | Persuasive communication (SCT). | At each visit, the dietitian discusses with participants how tracking their food intake helped them reach their goal and improve their eating patterns. |
| PO 6: Practice portion control by selecting healthy serving sizes  . | K1: Know recommended portion sizes for different food groups | Didactic instruction (SCT).  Active information processing (CLT).  Active information processing (CLT).  Didactic instruction (SCT)  Feedback (SCT). | Explain to participants the concept of “portion” in each food group and provide them with a written list of suitable portions for each group.  Explain to them how each portion can be measured using household items (a cup or a spoon) by using a visual guide to help them in the measurement process.  Inform them that daily portion quantities vary between genders based on the prescribed daily calorie intake.  Explain to the participant the importance of adhering to the daily portion quantities to control appetite and blood sugar levels.  Engage the participant in exchanging portions within the same food group (e.g., one apple or ½ of an orange juice).  Provide the participant with immediate feedback on his choice. |
|  | K2: Know how to use the household items as a visual guide to estimate portions. |  |  |
|  | K3: Know that portions vary based on individual calorie requirements. |  |  |
|  | K4: Understand how portion control affects hunger, satiety, and blood glucose. |  |  |
|  | S 1: Know how to estimate portion sizes when eating | Skills training (SCT).  Feedback (SCT) | Let the participant estimate portion sizes using food models or pictures during the session. Provide feedback and tips to improve accuracy. |
|  | S 2: Be able to use visual cues or tools to guide portions |  |  |
|  | S 3: Know how to adjust the portion size based on the calorie needs, as indicated by the dietitian, and in response to variations in blood glucose levels. | Guided practice (SCT).  Feedback (SCT). | The dietitian uses the participants’ food recall to match it with their blood glucose levels.  She reinforces good practices and corrects the ones that are not. |
|  | A1: Believe that knowing serving sizes helps control weight | Persuasive communication (SCT) | The dietitian provides personalized feedback to participants, illustrating the impact of adhering to the recommended serving size on weight loss. |
|  | A2: Value portion awareness as a tool for control, not restriction | Active information processing (CLT) | Discuss with participants the idea that awareness equals empowerment, allowing them to eat what they want, but in moderation. |
|  | A3: Believe that adapting portion size to personal needs is essential for achieving health goals | Goal setting (SCT)  Feedback (SCT) | The dietitian asks participants to quantify and describe the portions needed to manage episodes of hypoglycemia and hyperglycemia, control satiety, and achieve weight loss.  Immediate feedback is provided on this matter. |
|  | A4: Believe that adjusting portions is a flexible, self-care routine. | Modeling (SCT). | Share with participants a brief success story about a participant who learned to control portion size and achieved success in managing his diabetes and achieving weight loss with small, efficient changes. |
| PO7: Identify foods that align with recommended dietary patterns (MD or LC) | K1: Know which foods are considered healthy or unhealthy within the chosen diet. | Didactic instruction (SCT).  Active information processing (CLT).  Feedback (SCT) | Introduce both healthy and unhealthy dietary items related to MD or LC, both verbally and in writing.  Ask participants to list some of them and provide them with immediate feedback. |
|  | SE1: Feel confident in identifying and choosing compliant foods | Guided practice (SCT) | Participants demonstrate confidence when selecting appropriate food items in various situations. |
|  | A1: Believe that knowing what to eat is essential to managing weight and blood glucose. | Persuasive communication (SCT). | By using visuals to enhance message credibility and impact, participants are shown how small changes can improve blood glucose and support weight loss. |
| PO8: Prepare meals using whole, minimally processed ingredients | K1: Know which ingredients to prioritize | Didactic instruction (SCT).  Active information processing (CLT).  Feedback (SCT). | Inform participants about the food ingredients to prefer (unsaturated fat, complex carbs) instead of unhealthy ones (sugar, saturated fat…), both verbally and in writing.  Ask them to list some of the good and some of the bad food items and provide them with immediate feedback. |
|  | S 1: Be able to prepare meals from basic, whole ingredients. | Guided practice (SCT) | Participants are required to use the food ingredients that were introduced to prepare their meals. |
|  | A1: like A1 for PO7 |  |  |
| PO9: Read food labels to compare nutritional quality | K1: Know how to interpret dietary facts, especially total carbohydrate, for glucose control | Didactic instruction (SCT).  Active information processing (CLT).  Feedback (SCT) | The dietitian will teach the participant how to read and interpret food labels and choose the appropriate ones that fit their dietary needs.  Examples are provided to participants, who will, in turn, choose the appropriate product based on its label.  Immediate feedback is provided. |
|  | S 1: Be able to use food labels to select healthier options. | Guided practice (SCT). | The participant should follow the steps taught to interpret the food label and choose the appropriate product. |
|  | A1: Believe that label reading is essential to managing health. | Persuasive communication (SCT) | The dietitian discusses with the participant some examples of real products commonly used by him to highlight the “hidden pitfalls” and show how reading food labels supports better choices for better health. |

| BO2. Express confidence and skills in preparing meals aligned with the prescribed diet | | | |
| --- | --- | --- | --- |
|  | **Change objective** | **Theoretical method- Relevant theory** | **Practical strategy** |
| PO 1: List the necessary steps to prepare a balanced diet aligned with the dietary guidelines. | K1: Understand the dietary guidelines. | Didactic instruction (SCT).  Active information processing (CLT).  Feedback (SCT). | Inform participants about the dietary guidelines that define a healthy, balanced diet. |
|  | K2: Understand the components of a balanced diet. |  | Ask the participant to list the components of the balanced diet previously introduced.  Provide immediate feedback. |
|  | K3: List the steps involved in meal planning. |  | The participant is asked to list the steps involved in meal planning.  The participant should provide an example of meals composed based on the principles taught.  Immediate feedback is provided. |
|  | S1: Be able to identify and select food that aligns with dietary guidelines. | Skills training (SCT).  Feedback (SCT). | The dietitian shows the participant many food items and asks him to identify which item fits his dietary patterns.  Provide him with immediate feedback. |
|  | SE 2: Feel confident in organizing balanced and culturally appropriate meals. |  | The dietitian asks the participant to prepare three balanced, complete meals that fit his cultural background.  Provide him with immediate feedback. |
|  | A1: Value the long-term benefits of well-planned meals on health and diabetes control | Reinforcement Through Direct Experience (SCT) | Show participants the benefits they obtain from choosing balanced meals, including weight loss, fat loss, improved blood glucose control, and a more optimal overall metabolic profile.  For this purpose, at each clinical visit, the participant will have his body composition assessed using bioelectrical impedance, and his blood will be tested once every three months. |
| PO 2: Give a sample meal plan for two days | K1: Know how to distribute meals and snacks throughout the day. | Didactic instruction (SCT).  Active information processing (CLT).  Feedback (SCT). | The dietitian presents a sample daily meal plan (based on MD or LC) that includes proper distribution of meals and snacks. Then, participants are asked to build their daily plan using the same principles, with guidance and feedback. |
|  | K2: Know how to use dietary guidelines to structure the meals (MD or LC). |  |  |
|  | A1: Believe that meal planning is achievable and worth the time and effort. | Reinforcement Through Direct Experience (SCT) | At each visit, the dietitian assesses the impact of the participant’s food intake on satiety cues, weight loss, and blood glucose levels, emphasizing the importance of adequate meal planning for overall health and well-being. |

| BO3: Achieve and maintain 10-15% weight loss to support blood glucose control, diabetes remission, and reduce metabolic complications (dyslipidemia and low-grade inflammation). | | | |
| --- | --- | --- | --- |
|  | **Change objective** | **Theoretical method- Relevant theory** | **Practical strategy** |
| PO1: Follow the prescribed low-calorie diet for the assigned duration | K1: Know the benefits of losing weight on health and blood glucose control. | Didactic instruction (SCT).  Tailored information  (SCT). | The dietitian on the first visit explains to the participant the benefits of losing weight (5-10%) on overall health, more specifically on blood glucose control.  She uses participant-specific data to simplify the information (for example, your A1c could drop by 1 point, and your blood lipids will improve…). |
|  | S 1: Be able to integrate the low-calorie diet with physical activity (PA) while self-monitoring food intake for an appropriate weight loss. | Self-regulation skills training (SCT).  Guided practice.  (SCT) | The dietitian helps the participant create a weekly action plan that integrates meal planning (low-calorie), PA (based on fitness level), and self-monitoring (e.g., food diary or app). The plan is reviewed and adjusted based on outcomes and identified barriers.  *The IPAQ questionnaire is used to track PA.* |
|  | A1: Believe that following good eating patterns is a long-term practice for better health | Reinforcement Through Direct Experience (SCT).  Persuasive communication.  (SCT) | The dietitian regularly discusses the participant's progress in lab values (e.g., HbA1c, lipid profile) and weight change, emphasizing how consistent eating patterns contributed to these improvements. This repeated feedback reinforces the value of long-term adherence. |
| PO2: Be physically active for a minimum of 150 minutes per week. | K1: Understand the benefits of PA on weight loss and glucose control. | Didactic instruction (SCT).  Tailored information  (SCT). | The dietitian explains to the participants the importance of PA on weight loss and glucose control.  She provides them with specific, personalized examples to relate the information and simplify it (e.g., PA will help you lose more weight and lower your blood glucose levels). |
|  | A1: Believe that regular PA is essential to losing weight. | Modeling (SCT).  Persuasive communication (SCT). | The dietitian shows the participant an example of an individual who does not exercise and another who does, explaining the differences between them and the impact on weight loss and blood glucose levels.  Then ask the participant to reflect on how the activity could be beneficial for his case. |

| BO4: Engage in at least 150 minutes of physical activity (PA) per week | | | |
| --- | --- | --- | --- |
|  | **Change objective** | **Theoretical method- Relevant theory** | **Practical strategy** |
| PO1: Set achievable and realistic goals for PA practice that fit with diabetes control | K1: Recognize the importance of regular PA in weight management and diabetes control. | Didactic instruction (SCT). | The dietitian explains to the participant the importance of PA for weight loss, for diabetes, and for its associated complications. |
|  | K2: Be aware of the recommended guidelines for PA duration and intensity. | Tailored information  (SCT). | The dietitian informs the participant about the optimal intensity, frequency, and duration of PA, taking into consideration each case separately. |
|  | SE 1: Be able to set realistic and personalized goals | Goal setting (SCT).  Feedback (SCT). | The dietitian set SMART (Specific, Measurable, Achievable, Relevant, Time-bound) goals for PA with the participant.  The participant is encouraged to track progress and adjust goals as needed.  The dietitian provides feedback when needed. |
|  | A1: Value “goal setting” as a tool for controlling weight, PA, and health issues. | Belief selection (HBM). | The dietitian helps participants reflect on their successes from previous achievements to reinforce the belief that setting small, realistic goals is worthwhile and sustainable.  As an example, this can be achieved by comparing the effect of weight loss on the reduction of blood lipids and glycemia. |
|  | A2: Understand that setting small, realistic goals is more sustainable than drastic ones. |  |  |
| PO2: Incorporate PA in daily routine. | K1: Recognize the importance of PA in the daily routine to control appetite and blood sugar levels. | Didactic instruction  (SCT). | The dietitian informs the participant about the importance of PA in regulating blood sugar and appetite and tells him about the necessity of being active daily.  The information is transmitted orally and in writing. |
|  | K2: Know how to adjust glycemia based on the intensity and duration of PA. | Tailored information  (SCT). | The participant is informed about the PA that suits them, and they are provided with all the necessary tools to control glycemia, both before and after exercise.  Example: If blood sugar levels are too low, it is better not to start exercising before eating. |
|  | S1: Know how to coordinate the food intake with the type and intensity of exercise. | Guided practice  (SCT) | The dietitian works with the participant to plan meals for the exercise based on its intensity and duration.  The participant gives her a list of snacks that can be eaten before or after exercise.  Immediate feedback is provided. |
|  | A1: Feel motivated to make active choices during the day (climbing stairs instead of taking the elevator…) | Enhancing self-efficacy  (SCT) | During the counseling session, the dietitian identifies some achievable active choices that the participant can integrate into his lifestyle.  Participants are encouraged to track their success and reflect on how confident and motivated they feel when completing them. |
| PO3: Monitor and address barriers to being active. | K1: Understand common barriers to PA. | Problem Solving  (SCT). | Engage participants in discussing barriers that may reduce PA. Explore practical solutions to help overcome these barriers while remaining active. Participants should recognize that barriers are common, but they can be addressed. |
|  | K2: Know practical strategies to overcome the barriers. |  |  |
|  | K3: Understand that barriers are common and manageable |  |  |
|  | SE 1: Feel confident in adapting and adjusting the goals when needed without giving up. | Problem solving  (SCT). | The dietitian discusses with participants the possible barriers they may face and works with them to find a solution to overcome those barriers.  At the next appointment, participants inform the dietitian about the corrective action, and if they fail to overcome the barrier, the goal is adjusted accordingly. |
|  | SE 2: Believe in their ability to follow the weekly plan despite a busy schedule. | Action plan (SCT).  Self-monitoring (SCT).  Feedback (SCT) | Participants are asked to plan the weekly PA. They are encouraged to select a tracking method (pedometer or calorie counting) to monitor their activity and to stay motivated.  The dietitian tracks the PA of participants using the IPAQ questionnaire and provides them with immediate feedback on their progress. |
|  | SE 3: Ability to stay motivated and track progress regularly |  |  |

| BO5: Manage stress to support dietary adherence and emotional well-being. | | | |
| --- | --- | --- | --- |
|  | **Change objective** | **Theoretical method- Relevant theory** | **Practical strategy** |
| PO1: Utilize a coping strategy when experiencing stress. | K1: Know the connection between stress and emotional eating. | Problem Solving (SCT). | The dietitian explores the concept of emotional eating—when individuals turn to food for comfort—and discusses its underlying causes with the participants. Together, they brainstorm stress as a significant reason for emotional eating, sharing common signs of stress they've encountered. The dietitian and participants collaborate to identify their stress triggers and develop effective coping strategies together. |
|  | K2: Know the common signs of stress. |  |  |
|  | K3: Know some evidence-based coping strategies (deep breath, PA, problem solving) | Guided practice  (SCT). | After discussing stress and its triggers, coping strategies are listed and adopted. |
|  | S1: Be able to recognize stress triggers and respond adequately | Action planning (SCT).  Self-monitoring (SCT). | When the participant encounters a stressful situation, they will attempt to apply a coping strategy previously taught during sessions and report back to the dietitian at the next follow-up visit. |
|  | A1: Believe that stress management is essential for maintaining behavior change | Self-reevaluation (TTM).  Feedback (SCT). | The participant successfully uses the coping strategy by sharing his testimonial with the dietitian. *During follow-ups, the dietitian monitors the participant's stress level using the “perceived stress scale.”* |
| PO2: Plan for PA to reduce stress | K1: Know that PA is an appropriate coping strategy to relieve stress by improving mood. | Didactic instruction (SCT). | When discussing coping strategies, the dietitian emphasizes the importance of exercise in relieving stress and increasing energy expenditure. |
|  | K2: Know how to integrate PA in stressful moments. | Active information processing (CLT). | In the counseling session, the dietitian helps the participant brainstorm practical ways to include PA when feeling stressed. |
|  | SE 1: Feel confident to engage in PA when feeling stressed. | Mastery Experience.  (SCT) | After integrating PA as a coping stress method, the dietitian encourages participants to reflect on their ability to combine the coping strategies, reinforcing confidence successfully. |
|  | A1: Believe that PA is a valid, effective, and sustainable way to relieve stress. | Value clarification.. | Consider the previous situations where PA was successfully used as a coping strategy and appreciate this practice over time. |
| PO3: When uncontrolled emotional eating occurs, choose low-calorie items. | K1: Know a list of low-calorie, self-satisfying items to be eaten when stressed | Didactic instruction (SCT).  Active information processing (CLT).  Feedback (SCT). | The dietitian discusses with the participant a list of low-calorie items to be eaten when emotional eating occurs.  The participant should be able to recall at least three items from the list that can be taken when stressed.  The dietitian provides him with immediate feedback. |
|  | K2: Know the difference between physiological hunger and emotional hunger. | Didactic instruction (SCT).  Active information processing (CLT).  Consciousness Raising (TTM). | The dietitian presents a comparison table highlighting the characteristics of physiological and emotional eating. The participant is then asked to analyze personal experiences and classify them as physiological or emotional hunger. |
|  | S1: Be able to select a low-calorie item in moments of stress. | Self-monitoring (SCT).  Guided feedback (SCT).  Problem solving (SCT) | During the follow-up, the participant is asked to recall any instances of potential emotional eating and discuss how he could recognize and manage this situation effectively.  He also mentions the type of food chosen or the copy strategy applied. |
|  | S2: Be able to identify emotions and triggers before reaching for food. |  |  |
|  | A1: View this behavior as a step toward self-regulation | Self-monitoring (SCT) | By regulating emotional eating as directed, the participant will represent a form of personal control and will develop self-regulation skills. |

| BO6: Get adequate sleep to support metabolic and behavioral regulation. | | | |
| --- | --- | --- | --- |
|  | **Change objective** | **Theoretical method- Relevant theory** | **Practical strategy** |
| PO1: Plan to sleep for at least 8 hours/night. | K1: Know the importance of adequate sleep to control glycemia (allow normal hormonal fluctuations) and lose weight. | Consciousness raising (TTM). | The dietitian explains to participants how inadequate sleep alters hormonal regulation (insulin, cortisol, ghrelin, and leptin) and leads to cravings, overeating, weight gain, and uncontrolled glycemia. |
|  | K2: Know the impact of sleep deprivation on craving and dietary decisions. |  |  |
|  | S1: Be able to set and stick to a regular bedtime. | Problem solving (SCT) | The dietitian discusses with participants the optimal time for each person to go to sleep. Together, they discuss the barriers that often prevent them from getting a good night's sleep and work to find suitable solutions to overcome these obstacles. This may include using a relaxation technique or another method of their choice.  *The dietitian tracks the sleep patterns of each participant by filling out the “Pittsburgh Sleep Quality Index”.* |
|  | S2: Be able to identify and manage barriers to sleep. |  |  |
|  | S3: Be able to use relaxation techniques before sleeping (ex, reading) |  |  |
|  | A1: Value sleep as an initial component for appropriate weight loss and blood glucose control. | Consciousness Raising (TTM).  Guided reflection (TTM). | The dietitian engages participants in a conversation about how poor sleep impacts mood, leading to cravings, increased energy intake, and altered blood glucose levels, before discussing the benefits of improved sleep.  After trying the previously proposed solutions, the participants are asked to compare their mental and physical health when they were not getting enough sleep to how they feel after addressing this issue. |
|  | A2: Value the importance of sleep for optimal physical and mental health. |  |  |
| PO2: Follow a healthy lifestyle, like being physically active and decreasing caffeine intake, to improve sleeping quality. | K1: Know evidence-based tips that improve sleep quality, such as regular PA, caffeine reduction, and consistent sleeping routines. | Active information processing (CLT) | The dietitian shares and explains tips for better sleep, including reducing caffeine after 3 PM, taking a walk in the afternoon, and avoiding screens before bed. Participants highlight the tips they're currently following, those they find challenging, and one tip they'll try in the coming week. |

| BO7: Adhere to prescribed oral anti diabetic medications | | | |
| --- | --- | --- | --- |
|  | **Change objective** | **Theoretical method- Relevant theory** | **Practical strategy** |
| PO1: Set an alarm or a specific time to take the medication | K1: Be aware of the importance of taking the medication consistently to prevent complications. | Consciousness raising (TTM) | During counseling, the dietitian explains how consistent medication adherence can prevent serious complications, such as neuropathy or kidney disease. She uses real-life examples to show how missing doses can affect blood glucose levels over time.  *The dietitian uses the MGL scale (Morisky-Green-Levine scale) to track medication adherence.* |
|  | K2: Understand the effect of weight loss on step-down therapy (reduction of oral antidiabetic medication) | Persuasive communication (SCT).  Modeling (SCT). | The dietitian emphasizes the benefits of weight loss by using real-life scenarios, in reducing medication dosage (step-down therapy) and improving glycemic control. |
|  | SE 1: Be able to stick to the medication regimen and adjust food intake accordingly. | Guided practice (SCT). | The dietitian reviews the MGL score at each session, praises successful adherence, and collaborates on overcoming barriers when needed. This helps build the participant’s confidence in their ability to manage their medication consistently**.** |
|  | A1: Value the role of medication to control glycemia alongside diet and PA. | Persuasive communication (SCT).  Modeling (SCT) | The dietitian compares a case of inadequate medication adherence with one of proper adherence, highlighting the benefits gained from following the medication plan. |
|  | A2: Believe in the importance of losing weight on step-down therapy and the resultant health and economic benefits. | Feedback (SCT).  Reinforcement through direct experience (SCT). | During follow-up visits, the dietitian evaluates the extent of weight loss and its effects on blood glucose levels and blood test results. This allows participants to see tangible evidence of the benefits of weight loss on their health, reinforcing their belief in its positive impact. Additionally, by reducing medical expenses, participants will further appreciate the advantages of losing weight. |

| BO8: Attend scheduled monthly follow-up dietetic sessions throughout the intervention. | | | |
| --- | --- | --- | --- |
|  | **Change objective** | **Theoretical method- Relevant theory** | **Practical strategy** |
| PO1: Schedule sessions in advance. | K1: Understand the importance of consistent follow-up for managing dietary intake and overall health. | Consciousness Raising (TTM) | During counseling sessions, the dietitian emphasizes the importance of regular follow-ups to identify dietary mistakes, reinforce progress, and facilitate timely adjustments to meal plans or medications. She shares examples illustrating better outcomes, like weight control and fewer symptoms, for participants who attend follow-ups compared to those who do not. |
|  | SE 1: Feel capable of fitting appointments into the personal schedule. | Persuasive communication (SCT). | The dietitian helps the participant review their week to find suitable times for counseling. They discuss challenges such as transportation and work hours, allowing the participant to choose a realistic time slot, which reinforces their confidence in making it work. |
|  | A1: Value proactive planning to maintain consistent care. | Consciousness raising (TTM).  Value clarification (HBM). | The dietitian emphasizes that planning for appointments, such as setting reminders and choosing low-stress times, helps prevent last-minute cancellations and promotes consistent health improvement. She underscores the importance of responsibility and preparation.  Participants will, on their behalf, cooperate with the dietitian to set a suitable plan and adhere to it. |
| PO2: Actively participate in the sessions. | K1: Understand the benefits of active engagement (asking questions, sharing progress, and challenges). | Elaboration (CLT). | The dietitian explains how actively engaging in sessions (e.g., asking questions, sharing challenges) helps tailor the intervention to individual needs, enhances problem-solving skills, and leads to better outcomes. She shares brief examples of participants who benefited from being involved. |
|  | SE 1: Feel confident in sharing your concerns with the dietitian. | Guided practice with reinforcement  (SCT) | During counseling sessions, the dietitian shows empathy and provides supportive feedback, helping participants feel safe and capable of expressing their concerns and discussing their challenges in managing weight. |
|  | A1: Value the follow-up as beneficial and empowering. | Persuasive communication (SCT). | The dietitian presents evidence that regular follow-up is linked to better outcomes and highlights how previous successful participants used these sessions to gain control over their weight, helping participants see follow-up as a powerful tool for their success. |
| PO3: Reschedule for missed sessions. | K1: Understand that missing a session is not a failure; rather, it is essential to reschedule and stay on track. | Consciousness raising.  (TTM). | The dietitian explains that missing a session occasionally is common and not a sign of failure. However, to maintain progress, it is essential to reschedule and continue with the plan. |
|  | S1: Feel able to manage unexpected barriers. | Modeling (SCT) | The dietitian shares real-life examples of how other participants successfully handled unexpected barriers, such as missing a session or having a disrupted routine, to help the participant feel capable of doing the same. |
|  | A1: Rescheduling as a responsive behavior. | Self-reevaluation.  (TTM) | The dietitian encourages the participant to reflect on how rescheduling a missed session helped them stay connected to their goals, emphasizing that adjustments are part of a successful journey. |

| BO9: Utilize the mobile app to support adherence during the maintenance phase. | | | |
| --- | --- | --- | --- |
|  | **Change objective** | **Theoretical method- Relevant theory** | **Practical strategy** |
| PO1: Review progress reports, feedback, or reminders generated by the app | K1: Learn how to use the app interface effectively. | Guided practice  (SCT).  Consciousness raising (TTM**).** | The dietitian provides a step-by-step explanation of the app interface, showing participants how to log meals, PA, and weight. She explains that logging data helps monitor personal progress, receive automated feedback, and identify trends over time. The rationale behind each function is clarified to ensure complete understanding of the app’s purpose and use. |
|  | K2: Understand the purpose of logging data (monitor progress, receive feedback, identify patterns). |  |  |
|  | K3: Know the importance of maintaining weight loss. | Consciousness raising (TTM). | The dietitian explains that maintaining weight loss is essential for sustaining improvements in blood glucose control and reducing the risk of metabolic complications. |
|  | SE 1: Believe that consistency in using the app supports long-term maintenance. | Verbal persuasion (SCT) | In counseling sessions, the dietitian reviews the participant’s logged data, highlighting positive trends and improvements. She reinforces the benefits of consistent logging and encourages the participant's ability to maintain this behavior. |
|  | S1: Be able to interpret app-generated data (progress reports, feedback, reminders) and use it to guide decisions about behavior. | Guided practice (SCT).  Feedback (SCT) | During follow-up sessions, the dietitian and participant review a sample progress report or feedback message together. The dietitian explains how to interpret trends and feedback, then asks the participant to identify what behavior could be adjusted based on the data. |
|  | A1: Value the role of self-monitoring in maintaining weight loss. | Value clarification (HBM). | During the counseling session, participants reflect on past experiences and consider the effect of monitoring on aligning with their personal goals for maintaining a healthy weight. |
|  | A2: Feel motivated to engage with the app regularly. | Guided practice (SCT). | The dietitian emphasizes that the app can be personalized by allowing users to set goals, reminders, and track their progress visually. She encourages participants to select features that suit them and views the app as a tool for self-empowerment, rather than just a tracking device. |

| BO10: Maintain diet and lifestyle changes during the maintenance phase for continuous blood glucose control. | | | |
| --- | --- | --- | --- |
|  | **Change objective** | **Theoretical method- Relevant theory** | **Practical strategy** |
| PO1: Continue with regular PA for a minimum of 150 minutes/week | K1: Know the importance of PA in weight maintenance and glycemia regulation | Consciousness raising (TTM). | At the beginning of the maintenance phase, the dietitian explains to the participant the importance of staying physically active to maintain fat loss and preserve the improvement in glycemia observed in the previous phase (weight loss phase). |
|  | SE 1: Feel confident to plan and maintain PA even in a busy schedule | Guided practice (SCT).  Coping planning  (SCT). | During the initial session of the maintenance phase, the dietitian, along with the participant, established a schedule for regular PA and collaboratively agreed on a strategy to accommodate PA on busy days. |
|  | A1: Value the PA as an essential component to support weight maintenance | Value clarification  (HBM). | The dietitian discusses with the participant his views on maintaining an active lifestyle. She asks him to consider the benefits of being physically active for his health. The participant reflects on how staying active during this phase can align with his personal goals. |
| PO2: Continue to monitor weight at home. | K1: Know how to measure the weight accurately. | Instruction-Self-monitoring  (SCT). | The dietitian explains to the participant how, when, and how frequently the weight should be monitored (wearing light clothing, early in the morning after voiding, and weekly). |
|  | S1: Be able to initiate corrective action when weight is regained | Guided practice (SCT).  Problem solving  (SCT). | The dietitian asks the participant how he can manage his weight gain. She guides him through a scenario of recent weight gain, asking him to identify causes and select realistic corrective actions. The participant should be able to identify and initiate corrective action. |
|  | A1: Value weight monitoring as a self-care and empowering practice that provides control over one's health. | Value clarification (HBM). | The dietitian asks the participant how regularly tracking their weight has helped them feel more in control of their progress. She invites the participant to reflect on how this practice contributes to their self-care and autonomy in managing health. |
| PO3: Continue with monthly follow-up with the dietitian via face-to-face interview or through the app | K1: Understand the benefits of regular follow-up for maintaining weight and controlling blood glucose levels. | Action planning (SCT) | The dietitian stresses the importance of attending monthly follow-up appointments to monitor changes in weight and blood glucose levels. She assists the participant in setting reminders for these sessions and explains how to submit progress updates through the app, as needed, between appointments. |
|  | SE1: Feel capable of booking a monthly session or using the app. | Guided practice  Problem solving.  (SCT) | At the beginning of the maintenance phase, the dietitian discusses any potential barriers the participant may face in booking follow-up appointments or using the app. They review the steps involved in booking an appointment, and the dietitian encourages the participant to attempt to book an appointment independently before their next session. |
|  | A1: Value monthly follow-up as a part of diabetes management | Value clarification (HBM) | After several follow-ups during the maintenance phase, the dietitian asks the participant to reflect on how the follow-ups (face-to-face or through the app) helped him stay in control of his weight and blood glucose fluctuations. |
| PO4: Check blood glucose regularly and respond to essential fluctuations | K1: Recognize the symptoms of hypo- and hyperglycemia. | Guided practice- Feedback  (SCT) | At the beginning of the maintenance phase, the dietitian engages the participant in a review of target blood glucose ranges, helps them recall symptoms of hypo- and hyperglycemia, and uses recent glucose logs to discuss how to interpret values and take corrective actions when needed. |
|  | K2: Learn how to correct acute fluctuations, including hypoglycemia and hyperglycemia. |  |  |
|  | K3: Know the target blood glucose ranges. |  |  |
|  | K4: Know how to interpret blood glucose values and when to seek help. |  |  |
|  | S1: Be able to correct blood glucose fluctuations should they occur. | Guided practice with feedback.  (SCT) | The participant practices listing the steps required to correct blood glucose fluctuations. The dietitian provides immediate feedback, reinforcing correct actions and offering guidance on corrections as needed. |
|  | A1: Be motivated to use blood glucose data to make informed decisions about diet, PA, and medication. | Self-monitoring with feedback (SCT) | During follow-up sessions, the dietitian reviews the participant's blood glucose records, diet, PA, and medication used to assess progress. This encourages the participant to reflect on patterns and how their choices impact reading. This helps foster motivation to use data for tracking. |
| PO5: Seek and utilize family support and cost-effective strategies to sustain dietary and lifestyle changes during the maintenance phase | K1: Understand how family involvement and support can improve adherence to dietary and lifestyle changes. | Facilitation through social support (SCT) | The dietitian explains to the participant how involving a close family member in her dietary and lifestyle changes can help her stay motivated and improve adherence to her plan. She gives examples of how family members can offer support. |
|  | K2: Identify affordable and healthy food options, as well as cost-saving practices | Active information processing (CLT).  Feedback (SCT). | Provide the participants with a list of healthy foods and snacks that can be consumed during the maintenance phase and are also cost-effective.  Ask the participants to list some of them and provide them with immediate feedback. |
|  | SE 1: Feel confident in communicating needs and involving family in meal preparation if needed. | Mastery Experience through Self-Reflection.  (SCT) | The dietitian asks the participant to reflect on moments when he asked for family support. He is to compare these moments with times he did not ask and determine which was better. |
|  | SE2: Believe in the ability to maintain a healthy diet on a budget. | Modeling (SCT).  Guided Practice (SCT), Verbal Persuasion  (SCT). | The dietitian presents a low-cost, healthy weekly meal plan using local foods, then helps the participant adapt it and identify three cost-saving practices to use at home. |
|  | A1: Value the role of family support and smart budgeting in maintaining long-term health. | Self-reflection/elaboration (SCT). | During counseling sessions, the dietitian asks the participant to reflect on the impact of smart budgeting and family support in meal preparation and diet adherence. |

| BO11: Respond effectively to relapse and return to target behaviors. | | | |
| --- | --- | --- | --- |
|  | **Change objective** | **Theoretical method- Relevant theory** | **Practical strategy** |
| PO1: Identify high-risk situations that may lead to relapse. | K1: Understand common triggers and high-risk situations that can lead to relapse. | Risk identification (HBM).  Self-monitoring (SCT).  Relapse prevention model (SCT). | The dietitian discusses common triggers and high-risk situations with the participant that may lead to dietary relapses, including emotional, social, and environmental barriers, and encourages the participant to reflect on which ones apply to their daily life. |
|  | K2: Understand strategies to prevent relapses (problem-solving planning, social support). | Relapse prevention model (SCT) | The dietitian discusses with the participant some strategies to prevent relapses and address barriers, while also planning and seeking social support as needed. |
|  | SE 1: Feel confident in the ability to recover and resume healthy habits. | Mastery experience (SCT) | The dietitian asks the participant to share a case of recovery after a relapse and then highlights the strengths and steps they used to recover. |
|  | A1: Value self-compassion and resilience over guilt. | Outcome Expectation + Verbal Persuasion  (SCT). | The dietitian shares a brief example of how occasional setbacks are normal and encourages the participant to reflect on a recent lapse without judgment. Together, they identify what was learned and how to move forward, reinforcing the value of self-compassion and resilience. |
| PO2: Implement effective strategies when faced with a high-risk situation | K1: Understand different types of coping strategies (problem-focused versus emotion-focused). | Self-Reflection + Verbal Persuasion  (SCT). | Together, the dietitian and the participant explore various coping strategies, distinguishing between problem-focused (e.g., planning, seeking solutions) and emotion-focused (e.g., relaxation, self-soothing) approaches. They discuss when each type might be helpful and reflect on which ones the participant already uses. |
|  | K2: Be aware of harmful coping strategies like smoking. | Cognitive awareness  (SCT). | While discussing coping strategies, harmful ones are highlighted, especially smoking. |
|  | SE1: Be able to maintain coping strategies in different situations. | Self-regulation & guided problem-solving (SCT). | During follow-ups, the dietitian asks the participant to reflect on instances where coping strategies were successful and those where they failed. Together, they discuss the causes of the failures. |
|  | A1: Value self-care and emotional regulation. | Self-reflection (SCT).  Outcome expectation (SCT). | Participants are asked to reflect on the coping plan they implemented, how it supported their emotional well-being, and whether it helped them feel more in control of their situation. They are then encouraged to consider how such self-care contributes to long-term health. |
| PO3: Monitor weight and dietary behaviors to prevent relapses. | K1: Recognize the importance of self-monitoring to avoid relapses and efficiently manage setbacks. | Outcome expectation  (SCT). | The dietitian informs the participant that self-monitoring is crucial for maintaining weight loss and controlling diabetes; it helps prevent relapses and manage setbacks. |
|  | SE1: Feel confident in tracking food intake and weight consistently. | Verbal persuasion  Mastery experience  (SCT). | During follow-up, the dietitian reviews the participant's food and weight records, highlighting how consistent tracking helped identify and manage relapses, and encourages reflection on how these skills build confidence in controlling weight and diabetes. |
|  | A1: Believe that self-monitoring improves dietary habits and health. | Mastery Experience  Outcome expectation  (SCT) | The dietitian helps the participant recall previous instances where self-monitoring led to improvements in their dietary habits or health. Together, they reflect on these successes to reinforce the belief that self-monitoring is valuable and practical. |

**Abbreviations used for theories**

CLT: Cognitive Learning Theory

SCT: Social Cognitive Theory

HBM: Health Belief Model

TTM: Transtheoretical Model
