# Supplementary material for: A randomized controlled trial protocol comparing low-calorie Mediterranean and low-carbohydrate diets for diabetes remission in individuals with type 2 diabetes in northern Lebanon: an intervention mapping–based approach
Source: Front Public Health. 2026 Apr 8;14:1787980. doi: 10.3389/fpubh.2026.1787980 (PMC13100829; doi:10.3389/fpubh.2026.1787980)
Supplement: Supplementary file 3 [file Supplementary_file_3.docx]

**Supplementary material- S 3**

**Matrix of change objective- Step 5**

| **Determinants** | | | | |
| --- | --- | --- | --- | --- |
| **Performance objectives (PO) for Dietitians.** | **Knowledge** | **Self-efficacy/Skills** | **Perceived feasibility** | **Attitude** |
| **PO1: Attend and actively participate in the program training sessions.** | K1: Understand the purpose and components of the program | S1: Be able to apply training content to prepare and deliver counseling sessions as planned.  SE1: Feel confident in their ability to learn and apply the training content |  | Believe that training is relevant and will enhance program application and increase counseling effectiveness. |
| **PO2: Plan and hold counseling sessions in accordance with the study protocol.** | K1: Recognize the importance of scheduling the session in advance for better time management. | SE1: Feel capable of leading the sessions while maintaining the program integrity. | P1: Perceive the program as realistic and aligned with the current clinical practice |  |
| **PO3: Use tracking tools and mobile apps consistently to monitor patient progress throughout the study.** | K1: Understand the timing, purpose, and procedures for using tracking tools and mobile apps during and after sessions.  K2: Understand the features of the mobile app used in the maintenance phase to effectively teach students and utilize it adequately. | S1: Be able to use the tools as directed.  SE1: Feel confident using the tracking tools and mobile app for patient follow-up and documentation | P1: Perceive that using the tracking tools and mobile app is manageable within routine counseling workflows. |  |
| **Performance Objectives (PO) for adopters** | **Knowledge** | **Self-efficacy/Skills** | **Perceived feasibility** | **Attitude** |
| **PO1: Physicians refer eligible patients to trained dietitians.** | K1: Understand the benefits of losing weight for diabetes remission and the importance of dietary follow-up for that purpose. | SE1: Feel confident in identifying suitable patients and initiating referral. | P1: Perceive that referring patients will not disrupt their clinical flow |  |
| **PO2: Dietitians communicate with referring physicians when needed for patient care coordination** | K1: Understand how coordination between physicians and dietitians improves patient adherence and outcomes. | SE1: Feel capable of maintaining communication throughout the study. |  | A1: Realize the value of multidisciplinary communication in diabetes care. |
| **PO3: Clinic directors allocate necessary resources (e.g., time, space, staff support) to support the implementation of the counseling sessions.** | K1: Understand the program's structure, the need for a private counseling space, and its duration. | SE1: Feel confident in managing the appointment schedule and availability of dietitians within the program. |  |  |
